# Imaging mass cytometry reveals generalised deficiency in OXPHOS complexes in Parkinson’s disease

**DOI:** 10.1038/s41531-021-00182-x

**Published:** 2021-05-12

**Authors:** Chun Chen, David McDonald, Alasdair Blain, Ashwin Sachdeva, Laura Bone, Anna L. M. Smith, Charlotte Warren, Sarah J. Pickett, Gavin Hudson, Andrew Filby, Amy E. Vincent, Doug M. Turnbull, Amy K. Reeve

**Affiliations:** 1 Wellcome Centre for Mitochondrial Research, Translational and Clinical Research Institute, Newcastle University, Newcastle upon Tyne, UK; 2 Wellcome Centre for Mitochondrial Research, Biosciences Institute, Newcastle University, Newcastle upon Tyne, UK; 3 Flow Cytometry Core Facility, Newcastle University, Newcastle upon Tyne, UK

**Keywords:** Cellular neuroscience, Parkinson's disease

## Abstract

Here we report the application of a mass spectrometry-based technology, imaging mass cytometry, to perform in-depth proteomic profiling of mitochondrial complexes in single neurons, using metal-conjugated antibodies to label post-mortem human midbrain sections. Mitochondrial dysfunction, particularly deficiency in complex I has previously been associated with the degeneration of dopaminergic neurons in Parkinson’s disease. To further our understanding of the nature of this dysfunction, and to identify Parkinson’s disease specific changes, we validated a panel of antibodies targeting subunits of all five mitochondrial oxidative phosphorylation complexes in dopaminergic neurons from Parkinson’s disease, mitochondrial disease, and control cases. Detailed analysis of the expression profile of these proteins, highlighted heterogeneity between individuals. There is a widespread decrease in expression of all complexes in Parkinson’s neurons, although more severe in mitochondrial disease neurons, however, the combination of affected complexes varies between the two groups. We also provide evidence of a potential neuronal response to mitochondrial dysfunction through a compensatory increase in mitochondrial mass. This study highlights the use of imaging mass cytometry in the assessment and analysis of expression of oxidative phosphorylation proteins, revealing the complexity of deficiencies of these proteins within individual neurons which may contribute to and drive neurodegeneration in Parkinson’s disease.

## Introduction

Mitochondrial defects accumulate in substantia nigra (SN) neurons with advancing age^1–3^. The loss of this vulnerable population of neurons causes the motor symptoms associated with Parkinson’s disease (PD), and impairment of mitochondrial function has been suggested to play an integral role in this neurodegeneration. This dysfunction is not simply limited to the interruption of ATP provision and calcium homeostasis, but also involves the interaction of other organelles and proteins in the regulation of mitochondrial biogenesis and mitophagy (reviewed in Chen, et al^4^.). Post-mortem studies using human midbrain samples have provided valuable information regarding the mitochondrial contribution to the pathogenesis of PD, including the description of mitochondrial oxidative phosphorylation (OxPhos) complex I deficiency^5^ and an accumulation of somatically acquired mitochondrial DNA (mtDNA) deletions^1,2^. The deletions present within each SN neuron are unique in terms of their size, location and heteroplasmy level^2^, and are associated with varying degrees of biochemical defects within individual cases and between different neurons. These studies have highlighted the importance of single-neuron analysis in modern histopathological studies to improve our understanding of the role of mitochondria in the selective loss of dopaminergic neurons in PD.

To improve upon the detection of mitochondrial defects using chromogen immunohistochemistry, we have previously employed a quadruple immunofluorescent (IF) assay on midbrain samples^6,7^. This technique allows quantitative analysis of the expression of OxPhos subunits at the single-neuron level. However, since this technique is limited to four protein targets due to fluorophore wavelengths, we were limited to assessment of two complexes (namely complex I and IV). Therefore, a detailed investigation of how changes in the expression of one OxPhos complex affects the expression of other complexes and whether deficiencies were present for multiple proteins was difficult to achieve within single neurons. To understand more about the mitochondrial defects that occur in PD, we have optimised a multiplex imaging technique which will facilitate the single-neuron investigation of complex signalling pathways and protein interactions.

Imaging mass cytometry (IMC) couples detection of isotopic masses by “cytometry by time of flight” (CyTOF) with a high power, low wavelength UV-laser to allow fast ablation of tissue sections labelled with metal-conjugated antibodies. Each metal isotope is sequentially detected based on their mass-to-charge ratio, allowing simultaneous detection of over 37 isotopes from a single tissue section. The current generated by each isotope is reflective of the expression of the target protein, with the retention of spatial information, the images assembled provide sufficient resolution (~1 µm^2^ = 1 pixel) for analysis at a single-neuron level (~550–1800 pixels per neuron)^8,9^. Compared to IF, these images have the added advantage of an increased signal-to-noise ratio in highly auto-fluorescent tissues^10^. Here, we report the first application of IMC on formalin-fixed, paraffin-embedded (FFPE) human midbrain tissue with a panel of eleven antibodies developed following previous IF studies^6,7,11^. With this study, we provide validation of IMC detection for both protein expression and neuronal cell-type classification, providing a comprehensive evaluation of the expression level of all five OxPhos complexes simultaneously within individual SN neurons. Importantly, we assessed whether OxPhos deficiency extends beyond involvement of complexes I and IV in PD and whether deficiencies in multiple OxPhos proteins is present within single neurons. A cohort of nine PD cases and four mitochondrial disease cases with POLG mutations were included. Despite a similar pattern of acquired mtDNA deletions^2^, the POLG cases show variable neuropathological changes in the SN and were included as a comparison to identify PD specific changes which may contribute to the degeneration seen in PD, that is often absent from this region in mitochondrial disease ^12,13^.

## Results

### Optimisation of IMC analysis on FFPE human midbrain section

**Fig. 1:**
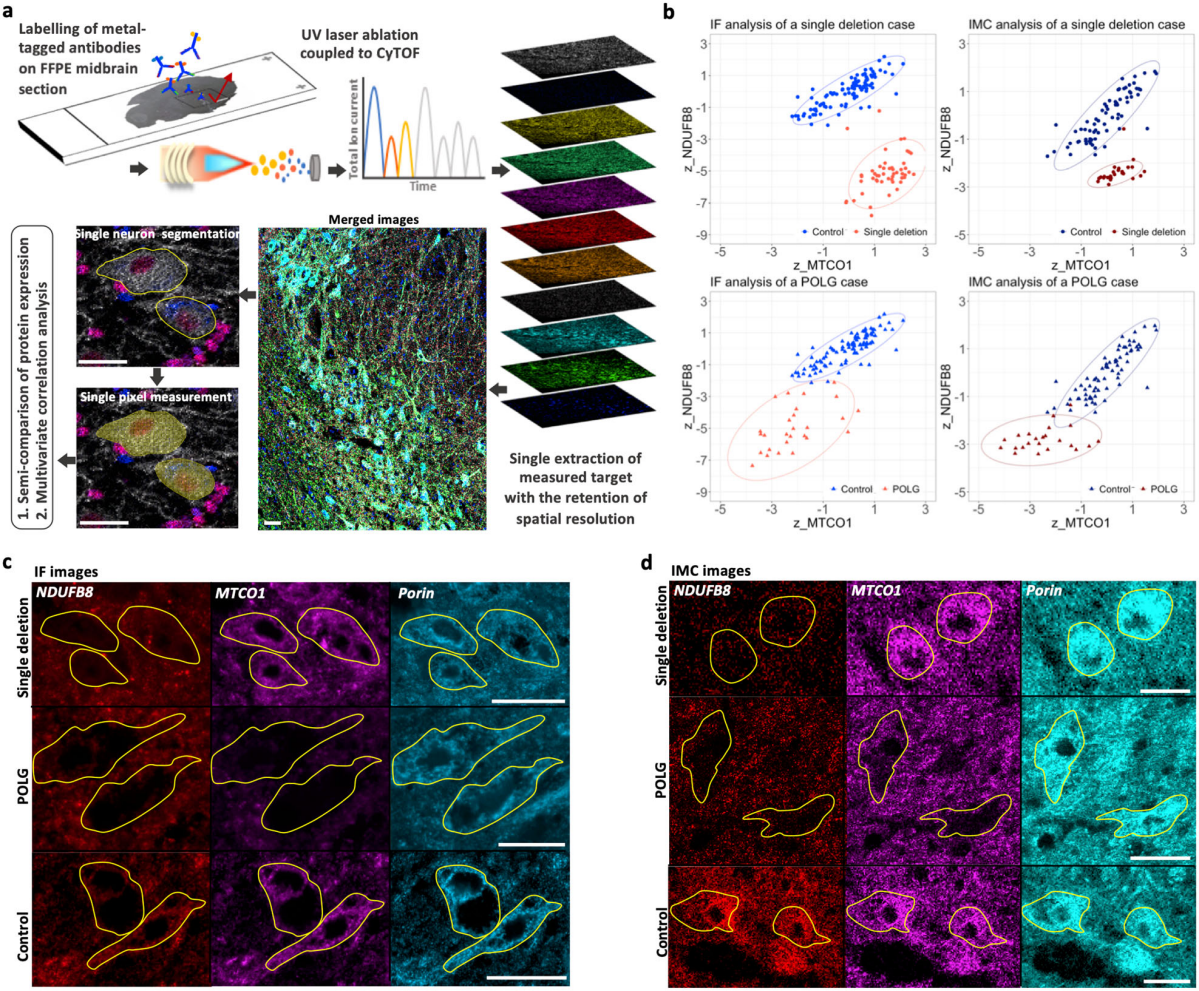
Workflow and validation of imaging mass cytometry (IMC) assay on human post-mortem midbrain. **a** A schematic demonstration of the IMC experimental workflow to investigate protein expression in individual midbrain neurons*.* Formalin-fixed, paraffin-embedded (FFPE) midbrain sections (5 µm) were immuno-labelled with twelve Lanthanide-metal-conjugated antibodies. Regions of interest within the substantia nigra (SN) region were ablated under a pulsed 213 nm UV laser beam, ionised and accelerated via an inductively coupled plasma source (ICP) of the “cytometry by time of flight” (CyTOF). Ion currents from each metal tag were detected as a signal intensity, reflecting the abundance of specific target proteins and assembled with spatial information into images. Individual tyrosine hydroxylase (TH)-positive dopaminergic neurons were manually outlined, with the single pixels within each outline automatically segmented. The mean signal intensity for only the cytoplasmic pixels per outlined neuron was calculated for further statistical analysis. **b** Validation of IMC profiling of mitochondrial oxidative phosphorylation (OxPhos) protein expression in the SN neurons by comparison to immunofluorescent (IF) data. Neurons from a mitochondrial disease case harbouring a single, large-scale mitochondrial DNA (mtDNA) deletion (red; female, 40 years) previously found to have an isolated complex I deficiency, and a patient with *POLG* mutations (dark red; male, 59 years, POLG03 in the cohort) which showed deficiencies in both complex I and IV (Chen et al^7^.). The *z* score data for complex I (z_NDUFB8) and complex IV (z_MTCO1) expression (normalised to VDAC1 expression) generated from both IF and IMC were plotted and compared to the same control cases (blue; 66 years and 73 years, male) respectively. The 95% confidence ellipse was also illustrated for each dataset. **c**, **d** Example images from IF and IMC. Individual neurons (outlined) negative for NDUFB8 and positive for MTCO1 from the single deletion case, are shown alongside neurons negative for both NDUFB8 and MTCO1 in the patient with *POLG* mutations, and both signals were normal in neurons from the control cases. Scale bar, 50 µm.

As a direct-detection technique for immunostaining, midbrain sections (5 µm) were labelled with primary antibodies directly conjugated to Lanthanide metal tags and IMC acquisition was performed as schematically described in Fig. 1a. For the validation of IMC, two cases were selected with previously characterised OxPhos complex deficiencies within SN neurons^7,12^. The first case showed decreased complex I but normal complex IV expression and harboured a single, large-scale mtDNA deletion (break point: *m.11657-15636*). The second case showed a high percentage of neurons with deficiencies in both complexes I and IV, and carried mutations in the POLG gene (*p.Gly848Ser and p.Ser1104Cy*s; POLG03). Data from IMC analysis showed comparable expression of the two proteins to those previously found using IF and demonstrated similar distribution patterns when complex IV (MTCO1) expression was plotted against complex I (NDUFB8) using z scores normalised to mitochondrial mass (VDAC1, Fig. 1b). Compared to controls, IMC data showed that 96.15% (98%, IF data) of neurons fell below the control range of complex I expression in the single deletion case. For the POLG case, 95.45% (96.15%, IF) of neurons showed expression of complex I which fell below the control range while this percentage was 54.55% for complex IV (45.15%, IF; Fig. 1b).

### Distinct individual variability in mitochondrial respiratory chain protein expression

**Fig. 2:**
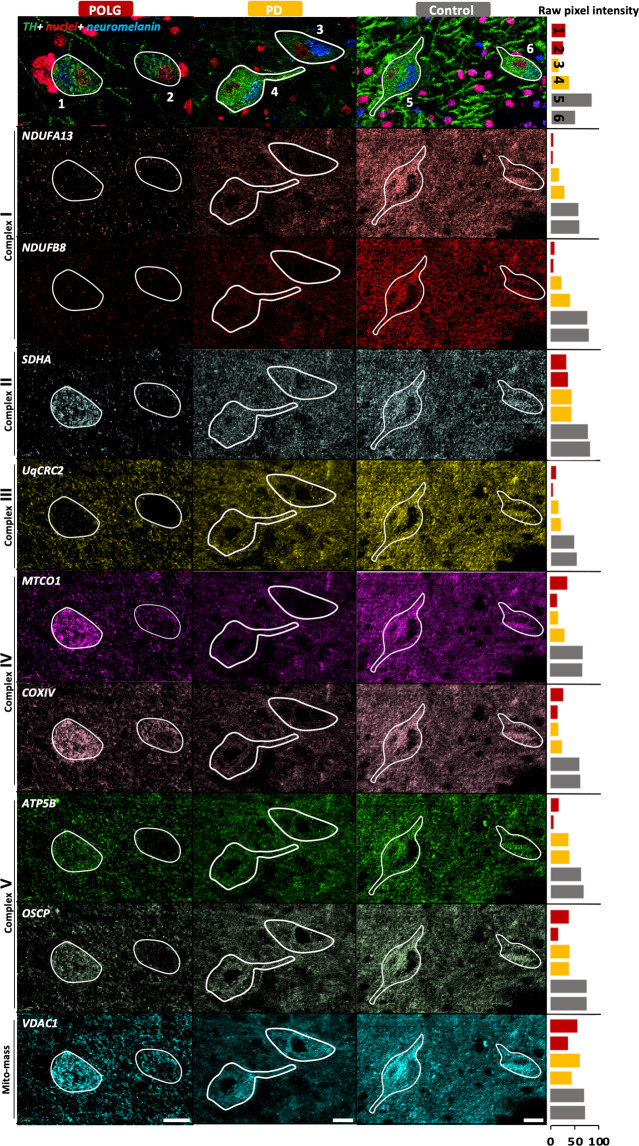
IMC images of TH-positive SN neurons. The signal from eight OxPhos complex subunits and a mitochondrial outer membrane protein (VDAC1) as mitochondrial mass marker were pseudocoloured to highlight mitochondrial protein expression. Representative images were selected from POLG02 (female, 23 years), PD02 (male, 70 years) and Con01 (female,18 years). Nuclei (red) are labelled by a Histone H3 antibody, neuromelanin (NM, navy) is visualised via the signal from Ir-intercalator binding. Each individual neuron was outlined based on TH-positive staining and intracellular presence of a nucleus and NM signal. Bar charts include to the right of each row of images illustrate the comparison of the raw mean pixel intensity per neuron, and the numbers on the bars refer to the neuronal number in the corresponding images (red-POLG; yellow-PD; grey-control). Scale bar, 20 µm.

Midbrain sections from patients with PD (*n* = 9), POLG mutations (*n* = 4) and controls (*n* = 10) were labelled with twelve metal-conjugated antibodies (Table 1). Example IMC images demonstrate abundant signal intensity for all tested subunits in the control SN neurons and varying degrees/combinations of signal loss in both patient groups (Fig. 2). For the control population, complex I subunits showed the weakest correlation with mitochondrial mass among the five OxPhos complexes (NDUFB8:VDAC1, Spearman *r* = 0.19, *p* < 0.0001; NDUFA13:VDAC1, *r* = 0.39, *p* < 0.0001). Complex IV subunit, MTCO1, one of the OxPhos subunits most affected by mtDNA defects^14^ also showed a weaker correlation with mitochondrial mass (*r* = 0.43, *p* < 0.0001), compared to the other subunits (*r* = 0.53–0.76, Supplementary Fig. 1). This can be attributed to decreased expression of these subunits within some SN neurons when compared to the majority of the control data, in line with the fact that the majority of the control cohort (8/10 cases) are aged over 60 years (Supplementary Table 1). Approximately 5–10% of SN neurons have been previously described as being complex I or IV deficient with normal ageing using immunohistochemistry^1,3,12^. Since the control dataset was not entirely normal, for comparative analysis, the lower limit of the 80% prediction interval of the control was used as an objective statistical limit to identify those neurons which showed a severe decrease in protein expression.

**Table 1:** IMC antibody panel for this study.

Antibody (Clone; RRID)	Target	Supplier	Purchase concentration	Conjugated metal tag	Working concentration
Anti-NDUFB8 (20E9DH10C12; AB_10859122)	Complex I (nDNA encoded subunit)	ab218218 Abcam	1 mg/ml	^160^Gd	0.04 mg/ml
Anti-NDUFA13 (6E1BH7; AB_10863178)	Complex I (nDNA encoded subunit)	ab218217 Abcam	1 mg/ml	^164^Dy	0.04 mg/ml
Anti-SDHA (2E3GC12FB2AE2; AB_301433))	Complex II (nDNA encoded subunit)	ab218213 Abcam	1 mg/ml	^153^Eu	0.02 mg/ml
Anti-UqCRC2 (13G12AF12BB11; AB_2213640)	Complex III (nDNA encoded subunit)	ab218215 Abcam	1 mg/ml	^174^Yb	0.02 mg/ml
Anti-MTCO1 (1D6E1A8; AB_2084810)	Complex IV (mtDNA encoded subunit)	ab218212 Abcam	1 mg/ml	^158^Yb	0.02 mg/ml
Anti-COX4+4L2 (10G8D12C12; AB_10862891)	Complex IV (nDNA encoded subunit)	ab218231 Abcam	1 mg/ml	^168^Er	0.02 mg/ml
Anti-ATB5B (3D5; AB_301438)	Complex V (nDNA encoded/ core subunit)	ab14730 Abcam	1 mg/ml	^170^Yb	0.02 mg/ml
Anti-ATP5O/OSCP (4C11C10D12; AB_10887942)	Complex V (nDNA encoded/ rotary stalk)	ab218232 Abcam	1 mg/ml	^161^Dy	0.02 mg/ml
Anti-VDAC1 (20B12AF2; AB_443084)	Mito mass marker	ab218214 Abcam	1 mg/ml	^166^Er	0.02 mg/ml
Anti-TH (TH-16; AB_477569)	Dopaminergic marker	SAB4200697 Sigma	1 mg/ml	^176^Yb	0.01 mg/ml
Anti-Histone H3-^171^Yb (D1H2; AB_2811058)	Nuclear marker	3176023D, Fluidigm	0.5 mg/ml	/	4 µg/ml

**Fig. 3:**
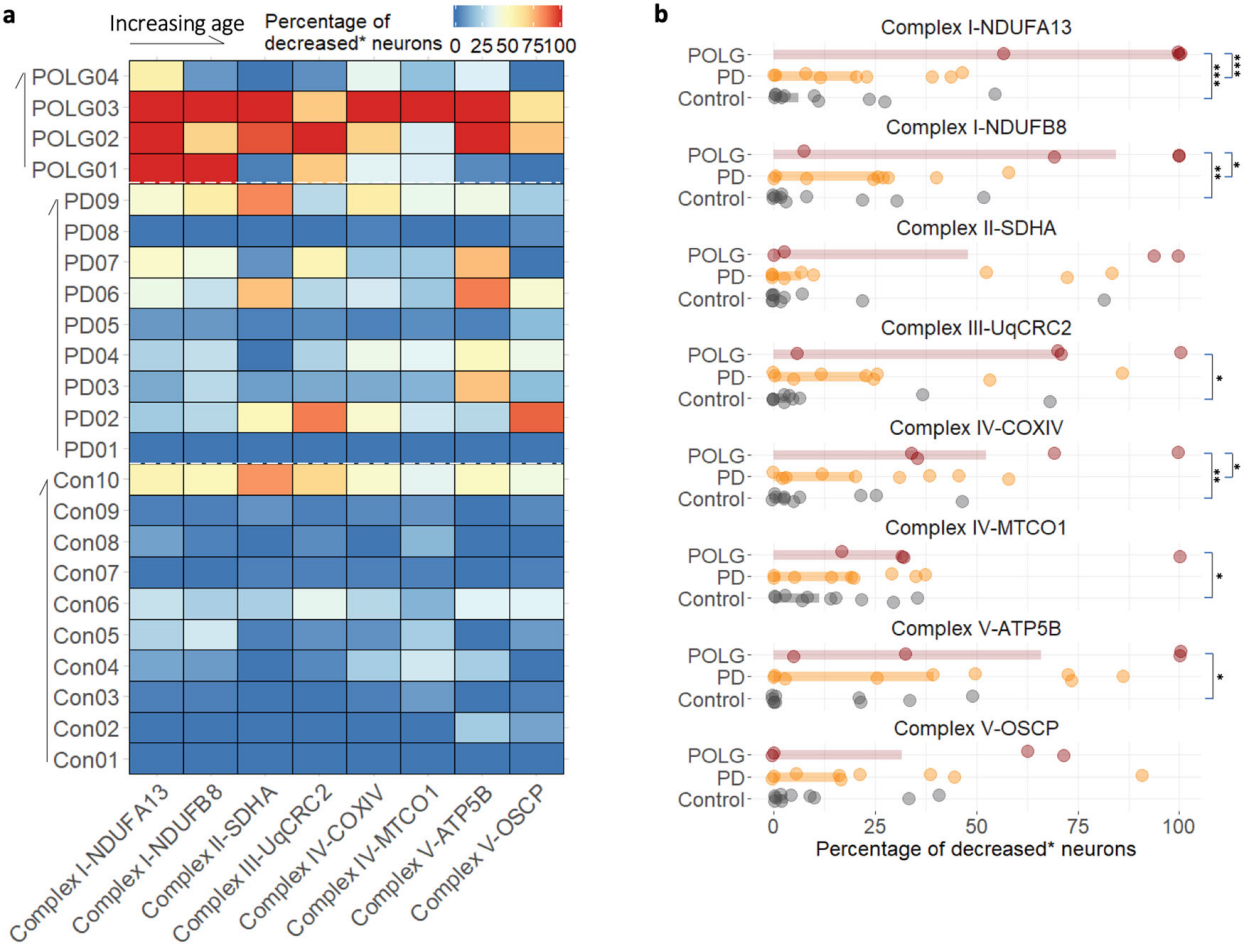
Profiling of mitochondrial OxPhos protein deficiency in disease and control cases. The percentage of neurons showing a decreased level of protein expression (*decreased-below the lower limit of the 80% prediction interval of the control regression) was calculated for each individual case. This percentage was presented for each protein target in a heatmap (**a**) and compared between groups (**b**). Bars show the median per group. The difference within each pair was analysed using a Mann–Whitney test (*p* value, *<0.05, **<0.01, ***<0.001).

In general, OxPhos protein expression was most affected in POLG mutation carriers as determined by analysis of the proportion of neurons showing a statistically decreased level of protein expression (Fig. 3a). However, given the heterogeneous nature of both PD and mitochondrial disease, the detailed characterisation of OxPhos protein expression for each individual case is an important investigation. In this study, we confirmed that mitochondrial OxPhos protein expression is variable between individuals, with varying combinations of affected subunits between cases. For example, complex I deficiency was the most prevalent defect identified in POLG01, however all five complexes were severely affected in POLG03. It is intriguing that the latter case also demonstrated Lewy body pathology, SN neuronal degeneration and Parkinsonian symptoms. POLG03 carried mutations (p.Gly848Ser and p.Ser1104Cys, Supplementary Table 1) situated in the polymerase domain of the POLG protein. Interestingly, other individuals who harbour POLG mutations involving the same domain have been reported to show parkinsonian features^15,16^, suggesting the susceptibility of the dopaminergic nigrostriatal system to mtDNA replication defects. In contrast, expression of OxPhos complexes was found to be preserved in the oldest POLG case, POLG04 (79 years), alongside a relatively intact SN neuronal population ^12^.

Neurons with decreased complex I or IV expression were identified in 7/9 PD cases (complex I: NUDFB8, 8.51–58.14%; NDUFA13, 7.89–46.67%; complex IV: MTCO1, 5.26–37.21%; COXIV, 2.63–58.14%; Fig. 3a, b). Interestingly, a high percentage of neurons were categorised as showing decreased expression of complex V in some PD cases (ATP5B: 4/9 cases, range 39.53–86.11%; OSCP: 3/9 cases, range 38.46–90.91%), though the disparity between expression of the two subunits in three PD cases (PD02, 03 and 07) requires further investigation. More than half of the neuronal population also showed decreased SDHA (complex II) expression in three PD cases (PD02, 07 and 10; range 52.27–83.72%). Overall, PD02 was found to be the most affected PD case with all five complexes showing decreases, in contrast, PD01 and PD08 were shown to have ‘normal’ OxPhos protein expression (Fig. 3a).

### Mitochondrial respiratory chain expression is less affected in PD neurons than POLG neurons

As previously reported^7^, complex I was the most commonly affected complex in the POLG patients (median and Interquartile Range (IQR): NDUFA13, 100%, 10.85%; NDUFB8, 84.38%, 46.55%; Fig. 3b). In the POLG group, the percentage of OxPhos decreased neurons was higher than the control group for all subunits, with the exception of SDHA and OSCP (Mann–Whitney test, *p < 0.05, **p < 0.01, ***p < 0.001; Fig. 3b). Complex I deficiency has been well-characterised in SN neurons of PD patients^6,7,17^, however, this study confirmed that such deficiency is significantly less common than in the POLG cases (NDUFA13, *p* < 0.01; NDUFB8, *p* < 0.001). For complex IV defects, particularly with regards to the expression of the mtDNA-encoded subunit, MTCO1, the distribution of PD and control cases was similar (median and IQR: PD, 19.44%, 24.29%; control, 11.20%, 16.21%; Fig. 3b). This suggests the presence of dysfunctional complex IV in aged neurons^1^ regardless of the occurrence of PD, which is associated with the age-related accumulation of mtDNA mutations^2^. In this present study, we also characterised PD and POLG cases which had a high proportion of complex II, III and V decreased neurons, yet such changes were not universal across either group.

**Fig. 4:**
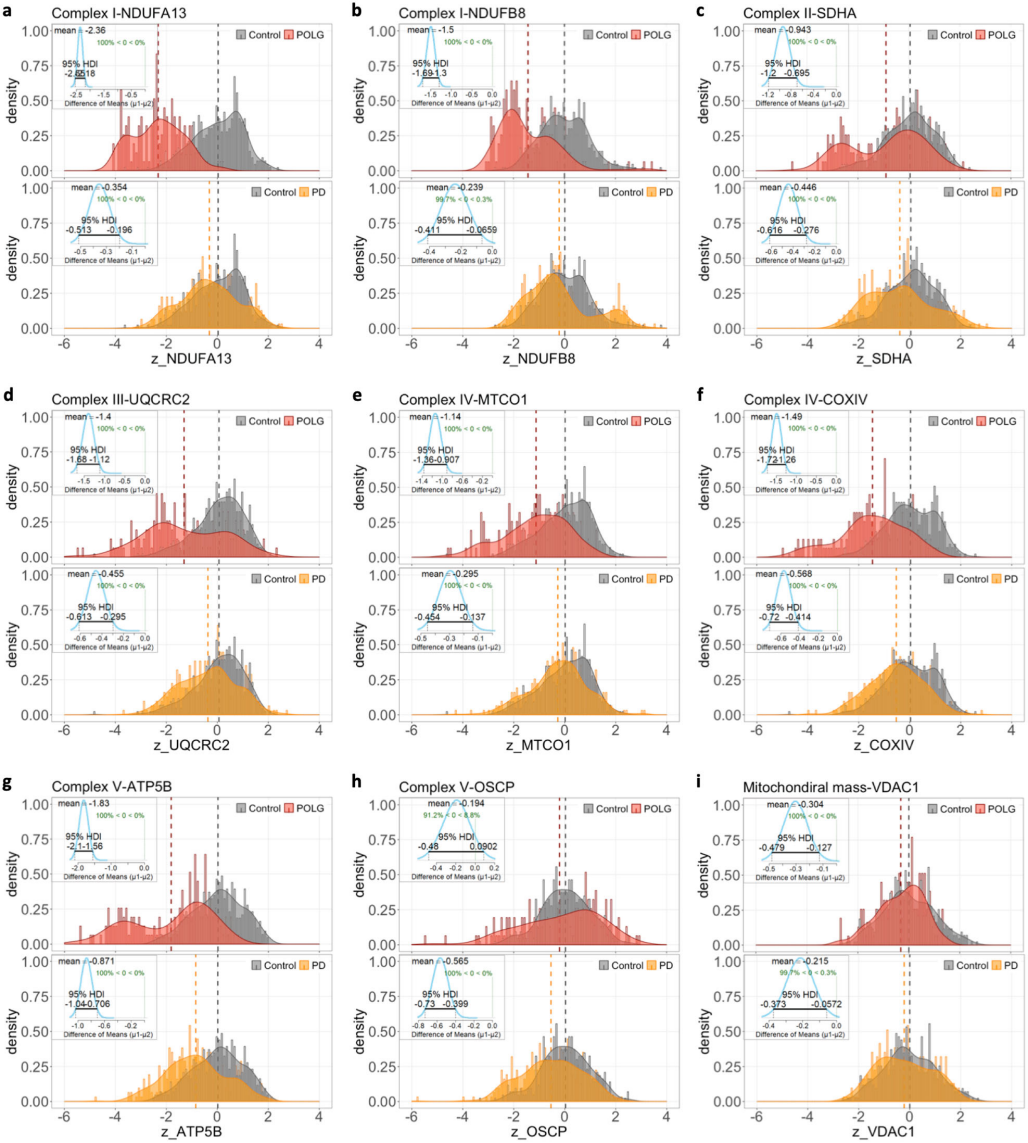
*Z* score distribution of OxPhos protein expression in single neurons from disease and control groups. **a**–**i** Frequency histograms (bin width = 0.1) demonstrate the comparison of the *z* score of tested OxPhos subunits (normalised to mitochondrial mass/VDAC1) and mitochondrial mass for individual neurons in two paired groups: POLG (red) versus control (grey), and PD (yellow) versus control. Number of neurons analysed: PD, *n* = 313; POLG, *n* = 156; control, *n* = 432). The density curve represents the normalised distribution pattern of each group dataset; the dash line shows the mean per group. The differences between every paired dataset were statistically described using the Bayesian estimation (BEST, (Kruschke^18^)). The insert shows the distribution curve of the mean of difference (*µ1–µ2*; *µ1*-diseased neurons, *µ2*-control neurons), its mean value and the 95% Highest Density Interval (HDI) output from the BEST analysis.

Next, individual neurons were grouped to compare the changes in OxPhos protein expression between each disease group and the controls, using *z* scores normalised to mitochondrial mass (Fig. 4). Six of the eight subunits present with a unimodal curve for the control group which is positively skewed, yet a multimodal distribution was seen for several plots from PD and POLG groups. This is particularly exemplified in the plots of NDUFA13, SDHA and ATP5B (Fig. 4b, c, g). These discrete distributions could be due to varying heteroplasmy levels for mtDNA mutations at the single-neuron level, alongside differences between individuals. It is also important to note that the contributed sample size from each individual varied, due to the prominent neuronal loss in some of the cases with Parkinson’s (Table 2). To aid the interpretation of the uncertainty between such datasets, Bayesian estimation^18^ was used to describe the differences between each paired group (Fig. 4, frames).

**Table 2:** The number of analysed neurons for this study.

	PD	POLG	Control
Case01	31	44	48
Case02	44	32	49
Case03	43	27	47
Case04	26	53	47
Case05	38	/	47
Case06	36	/	36
Case07	15	/	41
Case08	40	/	40
Case09	43	/	45
Case10	/	/	37
Mean	35.1	39	43.6
Standard deviation	9.6	11.7	4.7
Total number	316	156	437

In the Bayesian estimation models, the mean of difference (µ1–µ2) from each pair of posterior distributions represents the degree of difference between the two datasets^18^, and the higher its absolute value (|µ1–µ2|) with a narrower 95% highest density interval (95%HDI) indicates a more prominent difference. This analysis revealed that the expression of each of the studied OxPhos proteins was significantly lower in PD and POLG cases compared to healthy controls, within the exception of OSCP, which was only lower in the POLG cases. Of the five studied OxPhos complexes, the largest difference between the PD and POLG groups was identified for the expression of complex I (NDUFA13). Compared to the controls (µ2), the mean of difference for this protein was −0.36 for PD neurons with a larger 95% HDI (−0.51 to −0.20), yet this value was −2.36 for the POLG neurons (95%HDI, −2.55 to −2.18), suggesting the decrease in complex I expression is more severe in this group than in PD (Fig. 4). Similarly, the loss of MTCO1 was more severe in POLG neurons, compared to PD (*µ1–µ2*: PD, −0.295, vs. POLG, −1.14). In comparison, differences between the two disease groups were smaller in terms of the expression of SDHA (*µ1–µ2*: PD, −0.45 vs. POLG, −0.94) and ATP5B (PD, −0.87 vs. POLG, −1.83). Interestingly, changes in mitochondrial mass were also small in both disease groups compared to the controls (*µ1*–*µ2*: PD, −0.22 vs. POLG, −0.30).

### Widespread deficiencies across all mitochondrial respiratory chain complexes occur in PD

One of the key advantages of the IMC assay over IF is the simultaneous measurement of subunits of all five OxPhos complexes within individual neurons, we therefore further expanded our analytic strategy in order to achieve a more detailed profiling of associations between the expression of each complex and corresponding changes in mitochondrial mass. The severity of OxPhos deficiency within each neuron was assessed based on the number of complexes showing decreased expression (six categories: 0–5, Fig. 5a). Almost 50% of PD neurons and 74.62% of control neurons showed no OxPhos deficiency, with a trend for the proportion of neurons within each category to decrease as the severity of deficiency increased (fully OxPhos decreased: PD, 5.06%; control, 1.38%). However, neurons with *POLG* mutations did not show such a pattern; with 17.95% of POLG neurons identified showing a full OxPhos defect.

**Fig. 5:**
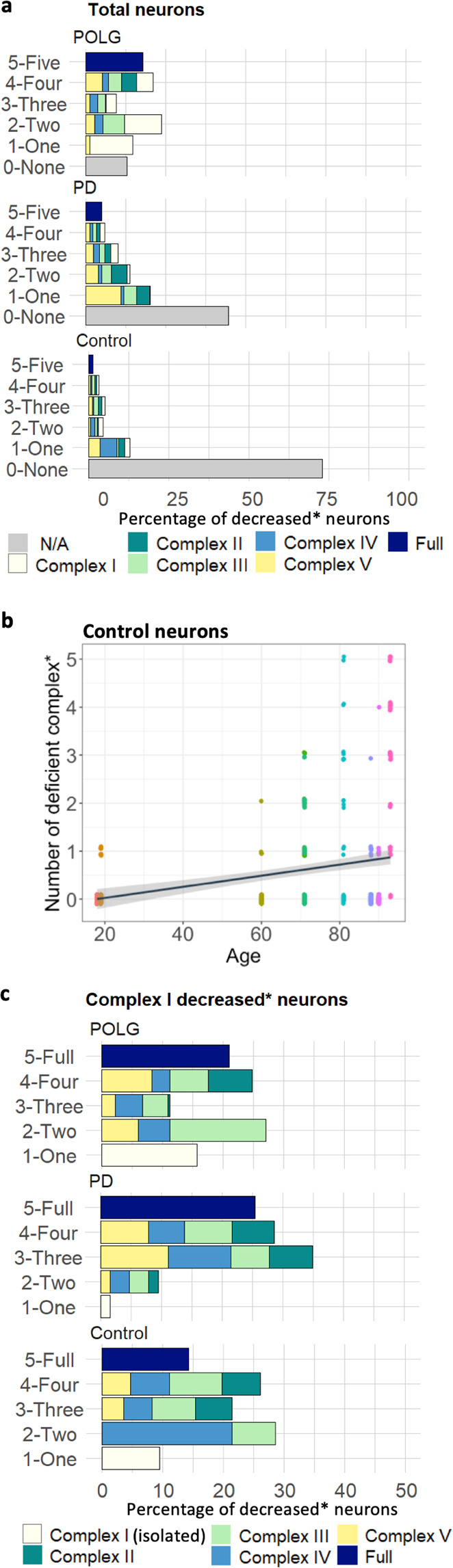
Investigation of combined mitochondrial RC deficiency and changes in mitochondrial mass in complex I decreased neurons. **a** Stacked bar charts show the percentage of neurons carrying different numbers of affected RC complexes that show a decreased level of expression in each group. Within each category, components of the ‘decreased’ neuronal population were colour-coded to show the proportion of each individual complex involved. For those complexes with two subunits tested, NDUFA13, MTCO1 and ATP5B were selected to represent the expression of complex I, IV and V respectively. Number of neurons analysed: PD, *n* = 63; POLG, *n* = 133; control, *n* = 42. **b** Regression analysis of the number of OxPhos complexes showing a decreased expression and age in the control cases (*r*^2^ = 0.07, *p* < 0.001). Each data point represents an individual neuron with separate controls highlighted by colour, for each neuron the number of proteins for which that neuron was classed as being deficient was determined. Lines and shadow represent the linear regression line and the 95% prediction interval. **c** Analysis of the percentage of neurons carrying different numbers of affected RC complexes that show a decreased level of expression in each group was also performed in the neuronal population with complex I decreased expression.

Previous studies have suggested that age may be one of the drivers of mitochondrial dysfunction, with prior evidence that respiratory chain deficient cells accumulate with age in many human tissues, often as a result of high levels of mtDNA mutations^1,19–21^. Our regression analysis also confirmed a significant positive relationship between age and the number of complexes showing decreased expression among the control cases (Fig. 5b; *r*^2^ = 0.07, *p* < 0.001), demonstrating that the severity of OxPhos deficiency could develop with advancing age in dopaminergic neurons. This is also supported by the fact that the expression level of all five complex subunits (with the exception of OSCP) showed a significant decline with advancing age (Supplementary Figs. 2–3).

As the largest OxPhos complex, deficiency in complex I has previous been implicated in the SN degeneration (reviewed in Chen et al^4^. and Reeve, et al^22^.). Our profiling analysis revealed that within the analysed dopaminergic neurons isolated complex I (NDUFA13) deficiency was rarely detected in PD neurons and most complex I decreased neurons harboured multiple OxPhos deficiencies (Fig. 5c), whilst for POLG neurons the most common isolated OxPhos deficiency was complex I (91.30% vs. 1.58%, PD). In PD, 28.75% of complex I decreased neurons showed decreases in all four complexes (POLG, 21.05%; control, 14.29%), and the most likely OxPhos defect detected involved two other complexes (34.92%; POLG, 11.29%; control, 21.42%; Fig. 5c). Despite a less significant loss of complex I expression in the neurons from PD compared to those with *POLG* mutations (Fig. 4a, b), the composition of the mitochondrial defect within these complex I decreased neurons in PD demonstrated a more complicated pattern than previously reported, particularly in terms of the combined loss of other OxPhos complexes.

**Fig. 6:**
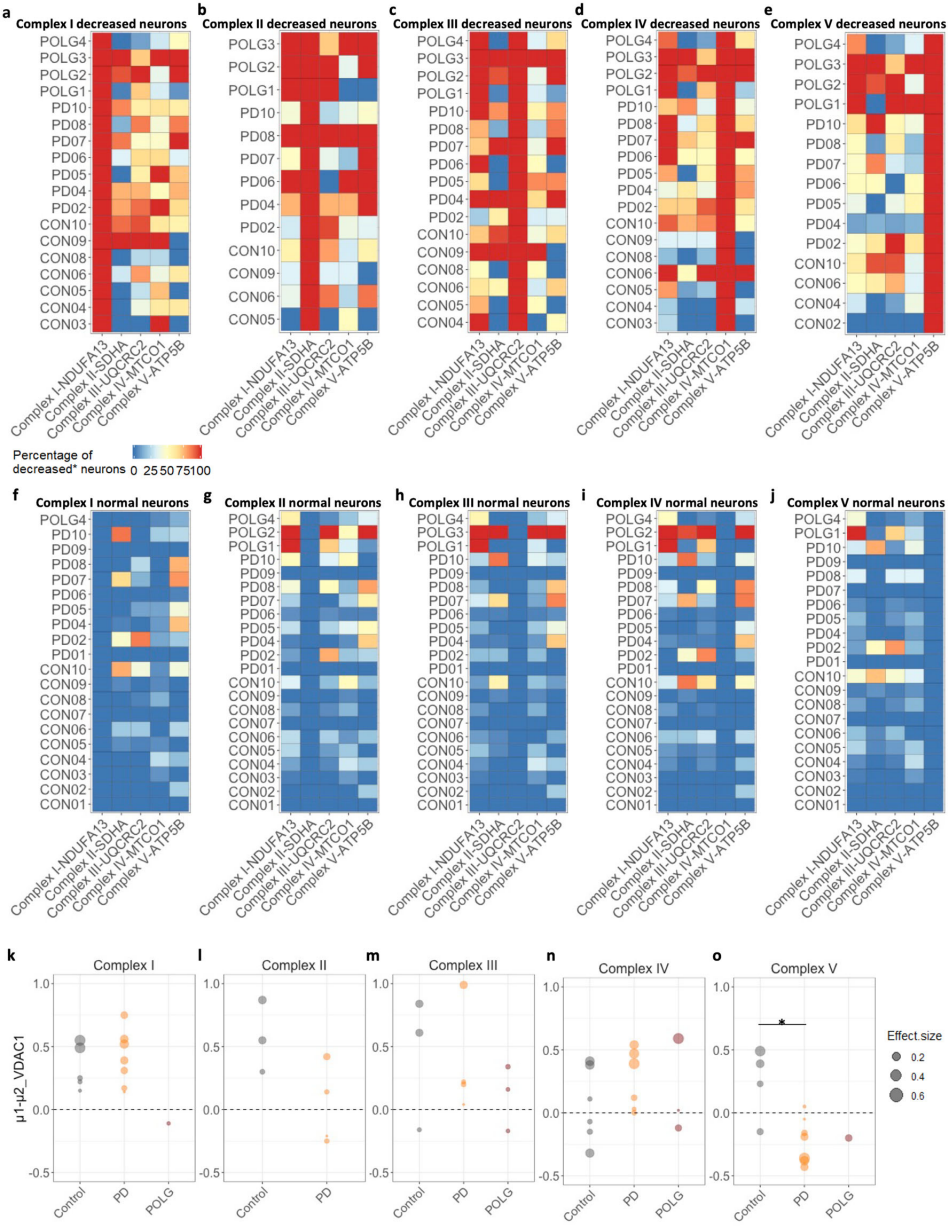
Complexity of OxPhos deficiencies within Substantia nigra neurons. **a**–**e** Heatmaps to illustrate the percentage of neurons which show a decreased level of protein expression within each individual complex among the five categories of deficient neuronal populations (complex I–V deficient neurons respectively). **f**–**j** These are shown alongside the same percentage calculations within the corresponding normal neuronal populations. Cases with no deficient neurons were excluded from this analysis. **k**–**o** Using Bayesian estimation, the difference of means (*µ1–µ2*; *µ1*-decreased, *µ2*-normal) of mitochondrial mass (VDAC1) between the decreased and normal neurons of each individual case was analysed and compared between groups. Only cases with three or more neurons which fell into each category were included. Group comparison of the *µ1–µ2* value of each individual was also performed (Mann–Whitney test, *p < 0.05). Dot sizes represent the values of the effect size.

Looking into each individual complex in more detail (Fig. 6a–j), all seven PD cases showed a varying yet high percentage of neurons with decreased complex III (42.86–88.89%; median, 66.67%), IV (42.86–100%; median, 63.16%) and V (33.33–85.71%; median, 80%) expression in the presence of complex I deficiency (Fig. 6a). In five out of the seven PD cases, more than half of the complex I decreased neurons also presented with a decrease in complex II (PD02-04, 07, 10; 60–100%). Surprisingly, in the complex I normal neuronal population, a severe decrease in complex II (45.71–85.71%), as well as complex V (62.50–77.27%) expression was often seen in these PD cases (Fig. 6f). While for the neurons which showed a decrease in complex IV (MTCO1; Fig. 6c), the proportion of neurons which showed an isolated decrease in this complex in PD cases was small (5.36%), and absent in POLG cases, in comparison to 39.29% in controls. Overall, these heatmaps demonstrate similar patterns within complex I, III, IV deficient neurons, confirming that in most PD cases and the two oldest controls, complex I, III and IV deficiencies are more likely to occur as a combined loss. (Fig. 6a, c, d). This could be due to the fact that these complexes closely interact in the process of electron transport^23^. Interestingly, in the complex V deficient neuron population (Fig. 6e), fewer PD neurons were observed which also have deficiency for the other complexes studied (complex I: 10.14–64.71%, median, 40.88%; complex III: 0–100%; median, 38.46%; complex IV, 13.29–61.54, median, 39.18%). This, again suggests that complex V expression could be lost independently of other OxPhos complexes in PD, particularly when compared to the POLG neurons.

All these data showed that three or more complexes were likely to be involved at the same time in neurons from both disease groups, leading to a more detrimental effect on OxPhos function and severe implications for neuronal survival compared to an isolated complex I/IV deficiency. Indeed, prominent loss of TH-positive SN neurons was determined in the two POLG cases with severe combined OxPhos deficiency (POLG02 and POLG03) and in 8/9 PD cases^12^ (Supplementary Fig. 4), with the exception of PD08 in which normal OxPhos protein expression was detected (Fig. 6a–j).

### Increased mitochondrial mass in complex I or IV decreased neurons: an ageing related effect?

Finally, we performed a comparison of the expression of VDAC1 between normal neurons and neurons with decreased OxPhos complex expression within individual cases to understand the changes in mitochondrial mass to OxPhos deficiency, using the Bayesian estimation (Fig. 6k–o). A significantly higher mitochondrial mass in the complex I decreased neurons compared to those showing normal expression was detected in all seven PD cases (effect size, 0.19–0.54) and 5/7 control cases (effect size, 0.20–0.71; Fig. 6k). For the analysed POLG case (POLG4), on the contrary, mitochondrial mass was lower in the complex I decreased neurons than those showing normal expression (effect size, 0.21). Similar analysis was performed between complex II, III, IV, and V decreased and normal neurons respectively (Fig. 6l–n). For complex III deficient neurons, a significant increase of mitochondrial mass was found in 4/7 PD cases (effect size, 0.21–1.11), 2/3 controls (effect size, 0.51–0.58) and 2/3 POLG cases (effect size, 0.17–1.29) was found (Fig. 6m). Complex IV deficient neurons show a significant increase of mitochondrial mass in 4/6 PD cases (effect size, 0.18–0.73), 3/7 controls (effect size, 0.51–0.58) and one POLG case (POLG01, effect size, 0.24; Fig. 6n). Whilst the level of mitochondrial mass was significantly decreased in the complex IV decreased neurons in Con03, 04, 08 and POLG04 case, compared to their complex IV normal population. These cases happened to have low numbers of neurons showing OxPhos defects compared to other cases within their groups (Fig. 3a). It indicates there may be a threshold of the degree of OxPhos dysfunction that contributes to the changes in mitochondrial mass, a consequence of altered balance between biogenesis and mitophagy. Such response was not detected in the neurons with a decrease in complex V (ATP5B) expression in the PD cases, leading to a significant difference of ‘the Difference of Means’ between the PD and control groups (Fig. 6o, Mann–Whitney test, *p* < 0.05).

## Discussion

Here we present the application of IMC for the multiplex analysis of mitochondrial OxPhos protein expression in FFPE human post-mortem midbrain sections. This platform provides a powerful tool for multi-array profiling of protein expression within single neurons allowing the study of relationships between protein expressions to be determined and permitting pathway analysis to be performed. Moreover, IMC can be combined with other techniques, for example RNA scope to provide comparative analysis between the proteome and transcriptome ^24^.

The selection of good quality antibodies is paramount to the success of IMC studies, both in terms of the quality of generated signal and their ability to detected deficiencies in expression, particularly following loss of function. To perform this in-depth profiling of OxPhos protein expression, we selected antibodies which have been previously shown to detect both nuclear and mtDNA OxPhos defects, have been previously validated and give good signal^7,11,25,26^. The reaction of these antibodies in immunoblotting, immunocytochemistry and immunofluorescence has been shown to correlate with the loss of protein when determined spectrophotometrically for complexes II and III^27^, and complexes I and IV^28^. Furthermore, these antibodies were also selected based on the strong link of chosen protein targets to mitochondrial OxPhos function. For example, we targeted the mid-later stage of complex I assembly (subunits NDUFA13/NDUFB8) which are known to associate with complex I function^29^. The MTCO1 antibody, targeted a complex IV subunit previously been shown to correlate well with Complex IV activity as determined by COX/SDH histochemistry^30^ and is linked to dysfunction caused by mtDNA major arc deletions, which are common in these neurons^1^. Finally, loss of OSCP has been found to lead to reduced ATP production^31^. Here we have used IMC to target a larger number of proteins than previously possible to assess deficiency in all five complexes, however, it would be possible to explore the full complement of subunits in an individual complex provided good quality commercially available antibodies could be identified. Furthermore, we acknowledge that as with any antibody-based assay the quantitative assessment directly measures protein level of the subunits in question and any impact on OxPhos complex function is indirect and made based on the functional evidence referenced above.

Alongside the detailed description of OxPhos alterations in SN neurons between individuals with PD, *POLG* mutations and controls, we highlight the variability in the expression of these proteins between age-matched individuals in each group. Within our cohort for example, two PD patients (PD01, 08) show intact OxPhos expression, with minimal evidence for neurons exhibiting a deficiency in any of the eight OxPhos proteins studied. Both cases had a relatively low Braak staging and PD08 in particular had a late age-of-onset. POLG04 which also shows relatively minor mitochondrial defects was the oldest POLG case in our cohort, but this individual was spared overt SN neurodegeneration^12^. The variations in expression of OxPhos proteins might suggest that different individuals have different capacities for OxPhos and can thus tolerate mitochondrial dysfunction to varying degrees. This hypothesis is applied with the caveat that although relatively well age-matched, the cohort of cases used in this study will have differing levels of pathology and degeneration (Table 3 and Supplementary Fig. 4). A study using a larger cohort would be able to further examine and determine such individual respiratory capacities. However, these differences in mitochondrial capacity might provide a hypothesis for what drives the vulnerability of these neurons to loss with advancing age, increasing the susceptibility of some individuals to PD. Such variability may be linked to mtDNA haplogroups^32,33^, for example haplogroup H has been linked with an increased risk of PD^34,35^ and higher intrinsic mitochondrial function in muscle compared to a composite group of individuals with different haplogroups^32^. Nuclear genetic factors also drive this variable respiratory capacity and have been found to contribute to phenotypic heterogeneity in patients with mitochondrial disease^36^. Therefore, differential OxPhos capabilities driven by variable expression and function of OxPhos proteins could result in the different thresholds between individuals for when decreased expression of these proteins will lead to mitochondrial dysfunction, ATP depletion, impaired neuronal function and loss.

**Table 3:** Summarized information for cases included in this study.

Group	Mean age (years)	Mean disease duration (years)	Mean PM delay (hours)	Mean Fixation time (weeks)	Sex
PD	79.78 ± 6.83 (68–90)	9.78 ± 3.03 (6–15)	38.67 ± 28.48 (7–88)	8.18 ± 3.62 (4.43–14.14)	9 Male
POLG	45.75 ± 28.06 (22–79)	17.5 ± 15.52 (5–37)	66.75 ± 24.52 (32–85)	9.78 ± 3.03 (7–19)	2 Female/2 Male
Control	67.9 ± 28.01 (18–93)	/	56.1 ± 20.95 (23–81)	14.50 ± 20.21 (5–71)	4 Female/6 Male

The use of IMC allows the complexity of OxPhos deficiency to be interrogated. To identify an expression signature of OxPhos proteins most relevant to PD, we compared SN neurons from PD cases to those from POLG cases. Individuals with *POLG* mutations often show high levels of mtDNA mutations within SN neurons and high levels of OxPhos deficiencies; however, in spite of this neurodegeneration in the SN is variable and often minimal^12^. Firstly, our data suggest a global reduction in mitochondrial mass, the expression of OxPhos proteins and thus function of individual mitochondria within surviving neurons in both PD and POLG. Using the approach outlined in this study, we reveal that the expression of subunits of all five OxPhos complexes show reduced expression in PD and POLG cases compared to controls, as highlighted by a shift in the distribution of expression profiles. These data suggest that there is a global reduction in the expression of the studied proteins in the two disease groups compared to controls as highlighted by the shift to the left in the expression profiles of each protein. Previous studies have often reported a widespread deficiency of complex I in PD which has been proposed to be the primary loss, with complex IV being lost later in the disease^7,17^. Here, we confirmed individual neurons in PD show reduction in the expression of complex I/IV, though the degree of reduction was less severe than in POLG cases. Importantly, we showed that neurons with a reduction in complex I expression in PD are often associated with a more severe phenotype compared to the other groups. Our data highlight that mitochondrial OxPhos deficiency in PD is more widespread, than being limited to just complexes I and IV and that the profiles of expression of OxPhos proteins in PD neurons are more similar to mitochondrial disease cases than controls. In PD neurons, the most common isolated deficiency was complex V core subunit ATP5B, with complex I deficient neurons often showing additional reductions in the expression of complexes IV and V. The severe combined deficiency with the inclusion of multiple complexes in addition to complex I is likely to have a more detrimental effect on mitochondrial function and therefore, neuronal survival in the PD patients. In POLG and control neurons where complex I deficiency is more likely to be isolated, or involve fewer complexes, there may be compensatory electron entry through complex II, or maintained membrane potential generation by complexes III and IV, this may ensure at least some residual ATP production which may be absent in PD.

As the only entirely nuclear-encoded complex within the OxPhos, isolated complex II deficiency has previously been linked to late-onset neurodegenerative changes in mitochondrial disease patients^37^. In this study, we also provide evidence for complex II deficiency in some PD neurons, above that detected in controls, but not as marked as in POLG cases. The mechanism which may drive the loss of complex II in the PD cases could be associated with various aspects of nuclear signalling^38,39^ or a functional degradation of complex II caused by the disorganised formation of supercomplexes (complex I, III, IV) previously reported in PD^38,39^. The OxPhos supercomplexes are believed to form as a compensative change to limit superoxide from dysfunctional complex I, resulting in increased stability of each complex and efficient ATP synthesis^23,40^. This is also consistent with the fact that neurons displaying decreased complex I expression from our PD cases also showed reductions in complex III and IV expression.

Interestingly, we identified a decrease in expression of mitochondrial complex V subunit, ATP5B in PD and some POLG cases. This was a reproducible finding in neurons which showed normal expression of complexes I/IV and most neurons with a reduction in these complexes. However, reduced expression of OSCP was not prevalent within PD neurons, which may reflect recent data suggesting that OSCP might be functionally distinct from the other core subunits of complex V and that ATP5B is more crucial for ATP synthesis^41^. This complex has previously been suggested to be upregulated in homogenate samples from the SN and frontal cortex of PD patients^42,43^. While more recently complex V has been suggested to be a target for alpha-synuclein, with this interaction initiating mitochondrial dysfunction^44,45^. The decrease in expression of an ATP synthase subunit within individual neurons may give further evidence of impaired ATP production in PD cases. However, the differences between ATP5B and OSCP expression requires further investigation, with analysis of more complex V subunits.

In addition, we provide evidence of compensatory increases in mitochondrial mass in SN neurons in response to complex I/IV deficiency, regardless of the differential baseline level of mitochondrial mass within each individual. Such compensation was mainly detected in the aged neuronal population (PD and controls). Given that both PD and POLG neurons show a similar pattern of multiple mtDNA deletions^2^, mtDNA copy number was found to be higher in PD neurons than those with *POLG* mutations^4^, explaining the potential of PD neurons to compensate for a certain degree of mitochondrial deficiency via an increase in mitochondrial mass. However, multiple lines of PD study have suggested impaired transcription of OxPhos genes in these neurons, including a reduction in TFAM expression^6,7^ and a low transcription level of PGC-1 responsive nuclear-encoded OxPhos genes^46,47^. Thus, highlighting that nuclear-mitochondrial signalling is likely to contribute to mitochondrial OxPhos deficiency and other aspects of mitochondrial biology. Our findings of complex V deficiency support an important role for altered metabolism signalling^48^. These warrant future IMC studies involving a large array of key signalling factors on top of the current twelve markers to help understand and identify the unique proteomic changes in PD.

To conclude, this study is the first to validate and use IMC for the multi-target proteomic analysis of FFPE midbrain tissue. Here we characterised mitochondrial OxPhos protein expression in detail within SN neurons, with a view to identifying patterns of deficiency which may provide insight into whether such deficiencies drive neurodegeneration in PD. We have shown marked variability in the expression of these proteins between individuals, which may reflect differential respiratory capacities. In addition, we interrogated the relationships between the expression of all five respiratory complexes and their deficiencies, showing that in PD compared to control and POLG cases OxPhos defects are often more severe and involve more complexes. This study extends our knowledge of the complexities of mitochondrial dysfunction in Parkinson’s and highlights the importance of understanding individual vulnerabilities. To further understand individual respiratory capacity and its links to neurodegeneration, studies with a larger sample size will be required with the inclusion of individuals with different haplogroups. The inclusion of additional mitochondrial disease cases, including those with mild or isolated OxPhos complex deficiency will allow the interpretation of OxPhos complex deficiency and its role in neuronal survival.

## Methods

### Tissue

FFPE human midbrain tissue sections were obtained from the Newcastle Brain Tissue Resource (https://nbtr.ncl.ac.uk/). Written consent for the use of all tissue had been given by the donors or next of kin and ethical approval was provided by the National Health Service Local Research Ethics Committee. The use of this tissue adhered to the Medical Research Council’s (MRC) guidelines on the use of human tissue in medical research. Clinically and pathologically diagnosed idiopathic PD cases (*n* = 9) and POLG cases (*n* = 4) were included, alongside individuals without neurological disease used as controls (*n* = 10) (Table 3**)**. We analysed all SN neurons that met our inclusion criteria. The number of neurons analysed per case from the collected the IMC images are as follows (mean ± standard deviation): PD, 35.1 ± 9.6; POLG, 39.0 ± 11.7; Control, 43.6 ± 4.7. Detailed information of the cases used for this study can be found in Supplementary Tables 1–2. While idiopathic PD is predominantly a disease of later life, patients with POLG mutations can experience symptoms much earlier in life^49^. Where possible, we sought age-matched controls for both disease groups and since some of our POLG cases were only in their 20 s, we obtained two younger healthy controls for comparison.

For the validation of IMC to IF analysis, midbrain tissue sections from POLG03 in the present cohort, alongside a mitochondrial disease case with a single-large-scale deletion (40 year, female) and two aged controls (66 years and 73 years, male) from a previously reported cohort^7^ were used.

### Antibody panel

Eleven antibodies were used in the IMC panel (Table 1) targeting nine mitochondrial proteins (all nuclear-encoded mitochondrial proteins, with the exception of mtDNA-encoded MTCO1), alongside a dopaminergic neuronal marker and a nuclear marker. For each complex the ratio of the number of investigated subunits to the total number are as follows; Complex I 2/45, Complex II 1/4, Complex III 1/11, Complex IV 2/13 and Complex V 2/27. These mitochondrial antibodies were selected based on consistent, specific signal from IF staining of human frozen muscle^25^ and FFPE brain^6,7,50^, immunoblotting of human fibroblasts (ATP5B and OSCP)^26^, previous detection of mitochondrial dysfunction caused by both nuclear and mtDNA mutations^7,11,25,26^. Selected antibodies were manufacturer customised in a PBS-only formula with the same clone targets as previous validations (Table 1).

To help minimise channel crosstalk, antibodies were ranked based on their IF signal strength in serial muscle fibre sections^51^ and conjugated to metal tags, in accordance to the optimal delivery of metal isotope to the mass detector. Conjugation of antibodies was performed using the Maxpar® X8 Multimetal Labelling Kit (Fluidigm) and tested on a CyTOF system (HeliosTM, Fluidigm) using UltraComp eBeadsTM (Thermo Fisher Scientific). The panel was also tested by IMC to ensure no prominent spillover was detected in the adjacent (±1) mass of isotope channels.

### Antibody labelling

FFPE midbrain sections (5 µm) were immune-labelled with the full panel (Table 1) using a protocol adapted from Chang, et al^52^. . Slides were incubated at 65 °C for 30 min, deparaffinised in Histo-Clear II (Agar Scientific) and rehydrated through an ethanol series (100%, 95% and 70% v/v) and ddH_2_O. Heat-induced epitope retrieval was conducted at pressure (Aptum Biologics) using Tris-EDTA solution (1 mM, pH8, Sigma) for 30 min, followed by 5% normal goat serum (NGS, Vector; diluted in DPBS) blocking. Each section was incubated with 50 µl of the antibody cocktail (prepared in 5% NGS) in a humidified chamber at 4°C overnight. Sections were washed in PBST (0.1% Tween-20 in DPBS, Sigma) and stained with Intercalator-Ir (312.5 nM in DPBS; 201192, Fluidigm) for 30 mins. After ddH_2_O washes, sections were air-dried for >30 min before processing for IMC.

### IMC image acquisition

IMC analysis of midbrain sections was performed on a Hyperion^TM^ imaging system coupled to a Helios^TM^ mass cytometer (Fluidigm). Prior to laser ablation, panoramic optical images of each section were obtained under a 20X objective lens within the Hyperion. Regions of interest (ROI) were determined according to the distribution of dopaminergic neurons within the SN, mapped in advance using tiled imaging (Zeiss AxioImager) based on positive TH labelling (IF) on a serial midbrain section (Supplementary Fig. 5). ROIs with a total area of 3–6 mm^2^ were ablated for each section. The acquisition of data from each ROI was carried out at a resolution of ~1 µm^2^ and at a laser frequency of 200 Hz. Ion current data for the metal tags were resembled with the retained spatial information from ablation, generating integrated greyscale images as.mcd files (MCD viewer 1.0.560.2, Fluidigm).

### Data analysis

Images generated from IMC were exported as tiffs (MCD viewer, Fluidigm) and converted to pseudocoloured images for visualisation using Fiji^53^ and QuPath 0.2.0^54^. All surviving individual dopaminergic neurons within the SN region were manually outlined using the following the criteria: positive TH signal, clear nuclear labelling, and the presence of intracellular neuromelanin detected through its propensity to bind the Ir-Intercalator. Single pixel segmentation was performed within each outlined neuron to filter out the pixels of the nucleus and neuromelanin (pixel intensity > 5), extracting the mean intensity for the cytoplasmic pixels for statistical analysis. For each case, the number of analysed neurons per case are listed in Table 2. Neighbourhood and cluster pattern analysis of midbrain neurons was not included due to separated ROI acquired for individual section (Supplementary Fig. 6).

Statistical analysis and graph generation was performed using R 3.6.1^55^. Using the natural log-transformed intensity value of individual neuron, the linear regression between each OxPhos subunit examined and VDAC1 was generated (Supplementary Fig. 1). *Z* scores for each neuron were calculated from the standard deviation (SD) of a straight-line distance from the data point to the control regression, using a published programme (http://mito.ncl.ac.uk/immuno/)^25^. This enabled the estimation of protein expression from the predicted level according to the mitochondrial mass. The calculation of *z* scores for mitochondrial mass was based on the mean and the SD generated from a normalised VDAC1 distribution from the control dataset^25^. A decrease in the level of mitochondrial subunit expression was statistically defined when the plotted data fell below the lower limit of the 80% prediction interval for the control regression (Supplementary Fig. 1). The statistical differences between each disease and control datasets were described using the Bayesian estimation (the BEST package in R)^18^ and Mann–Whitney *U* test. To allow the analysis of data sets containing different numbers of values, we used Bayesian estimation (which accepts the null value and takes the uncertainty of dataset into account) for the comparison between groups with different sample size, rather than using traditional *t* test^18^. Detailed output from the Bayesian estimation for this study can be found in Supplementary Tables 3–4.

### Reporting summary

Further information on research design is available in the Nature Research Reporting Summary linked to this article.

## Supplementary information

Supplementary figures and tables

Reporting Summary

## Data Availability

Raw dataset generated and analysed during the current study (for all figures and supplement materials) are available in the ‘Figshare’ repository (DOI: 10.25405/data.ncl.14079497). Data are available under the terms of the Creative Commons Attribution 4.0 International license (CC-BY 4.0).

## Code availability

The custom R code used to generate results that are reported in this study are available from the corresponding author upon reasonable request.
