# Supplementary material for: Imaging mass cytometry reveals generalised deficiency in OXPHOS complexes in Parkinson’s disease
Source: NPJ Parkinsons Dis. 2021 May 12;7:39. doi: 10.1038/s41531-021-00182-x (PMC8115071; doi:10.1038/s41531-021-00182-x)
Supplement: Supplementary file 1 — Supplementary figures and tables [file 41531_2021_182_MOESM1_ESM.pdf]

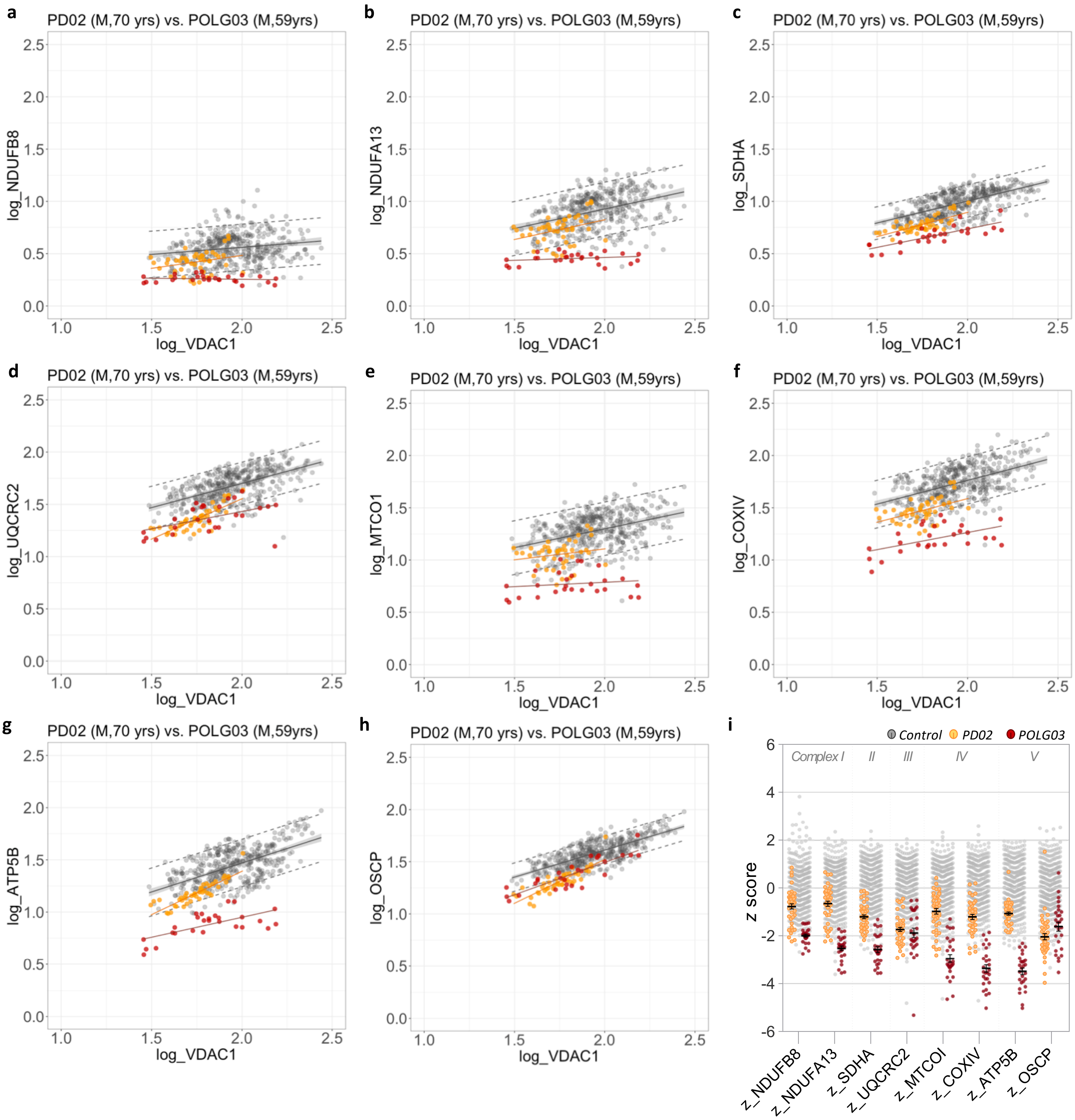

Supplementary Fig. 1. Comparative analysis of an example PD and POLG case. (a-h) Correlative analysis of protein expression in individual neurons from an example PD case (yellow; PD02, n=44) and a mitochondrial disease case with polg mutations (red; POLG03, n=27), relative to all control neurons (grey; n=432). Expression levels of each of the eight targeted subunits were plotted against mitochondrial mass. Solid lines show the linear regression in each dataset. For the control regression, the shaded area depicts the 95% confidence interval and the dashed lines show the 80% prediction interval of the regression. (i) The log-transformed data output from the IMC analysis was then transformed into z scores (which determines how many standard deviations away from the control regression trend each point sits), allowing the comparison of the protein expression level normalized by mitochondrial mass. Horizontal lines and bars show the mean and the standard error of the mean (SEM).

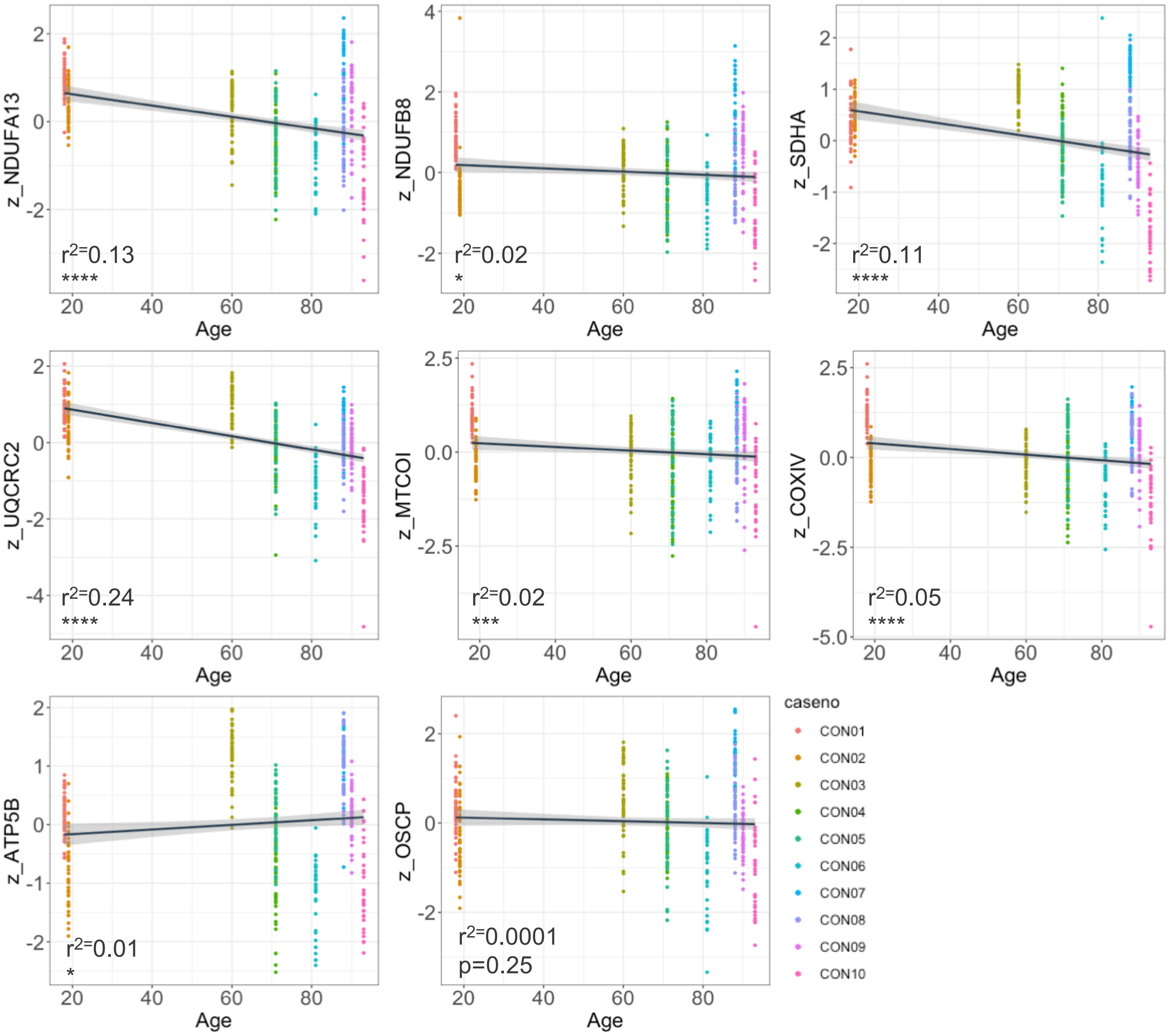

Supplementary Fig. 2. Relationship between age and expression level of each OxPhos protein in the controls. Each data point represents an individual neuron and each colour denotes a separate case. Lines and shadow represent the linear regression and the 95% prediction interval (simple fitted, Spearman rank correlation) respectively. Adjusted r^2 and p values (*<0.05,**<0.01, ***<0.001, ****<0.0001) for each pair of variants are illustrated on each graph.

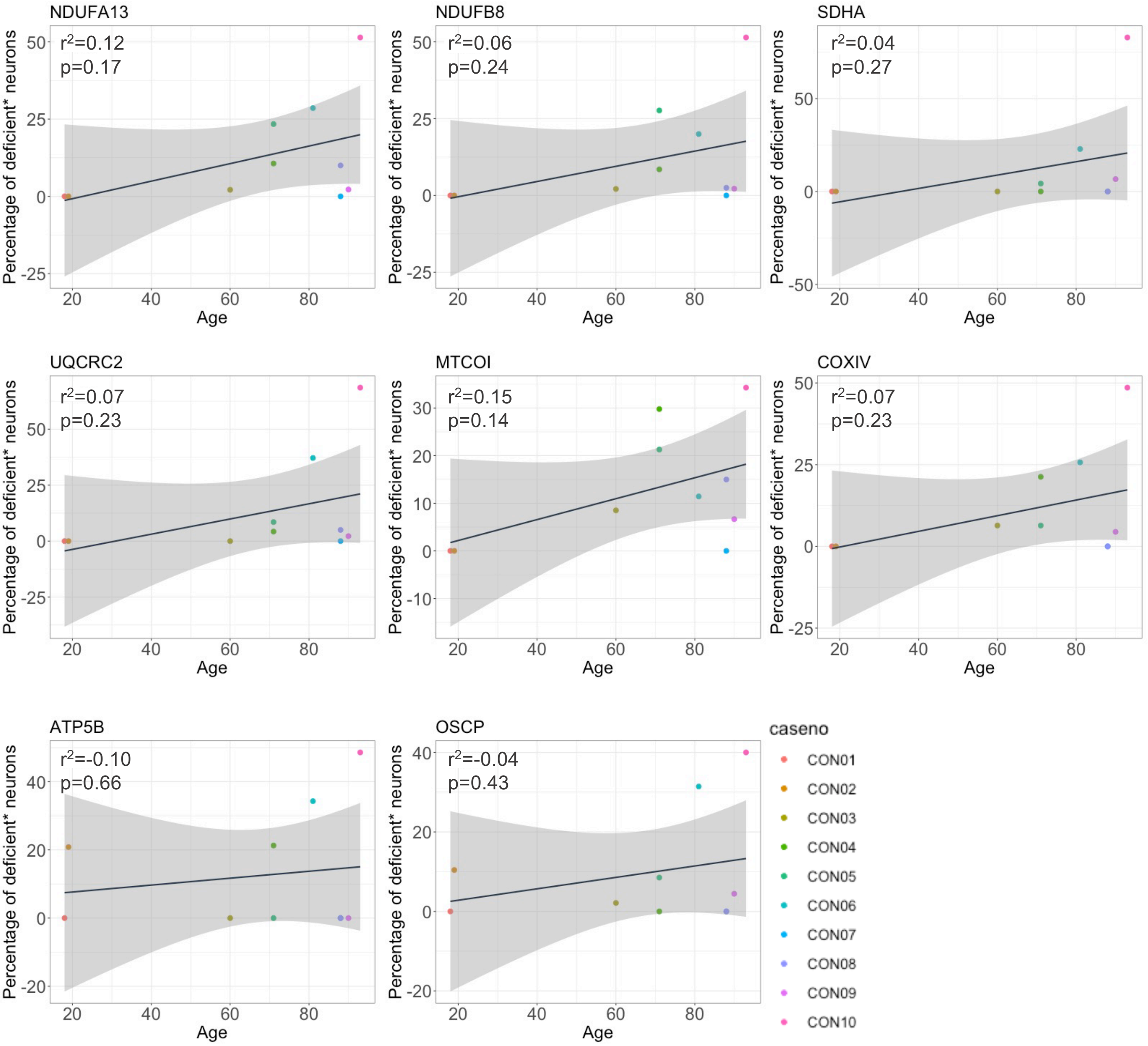

Supplementary Fig. 3. Relationship between age and the percentage of neurons deficient for each OxPhos protein in the controls. Each data point represents an individual control case, demonstrated with the adjusted r^2 and p value. Lines and shadow represent the linear regression line and the 95% prediction interval.

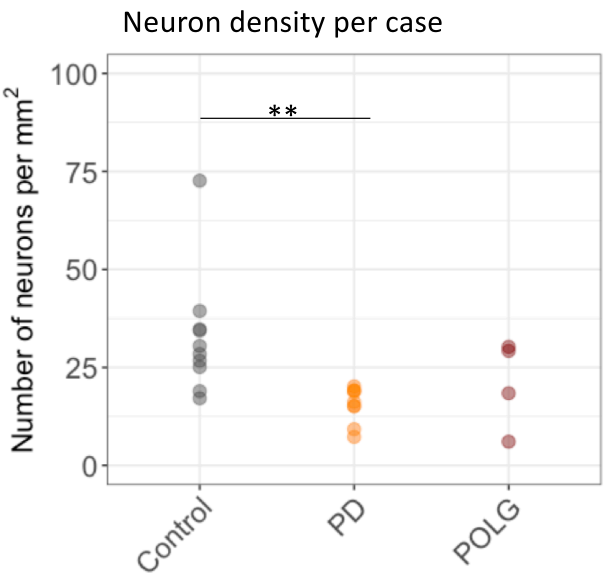

Supplementary Fig. 4. Calculation of the number of neurons from the acquired ROIs for each individual case. (One-way ANOVA, Control vs.PD, **p=0.01; Control vs.POLG, p=0.23).

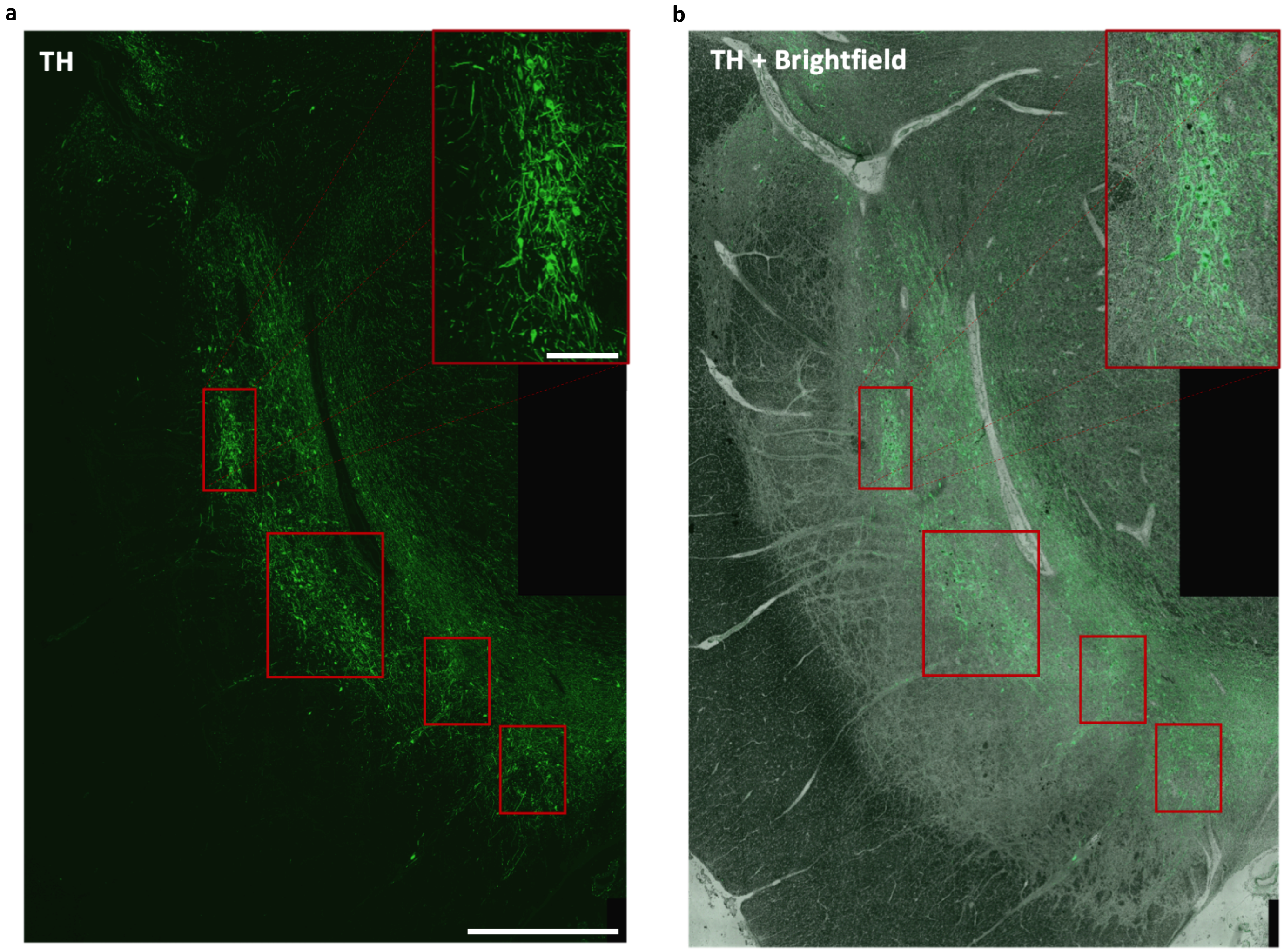

Supplementary Fig 5. Example tiled images of the SN region on the FFPE midbrain section. (a-b) Dopaminergic neuronal body and processes in an intact SN region were label with TH antibodies using IF. Three ROI with abundant TH-positive neuronal population were selected for IMC detection (Frames). Images were selected from Con06. Scale bar, 200uM.

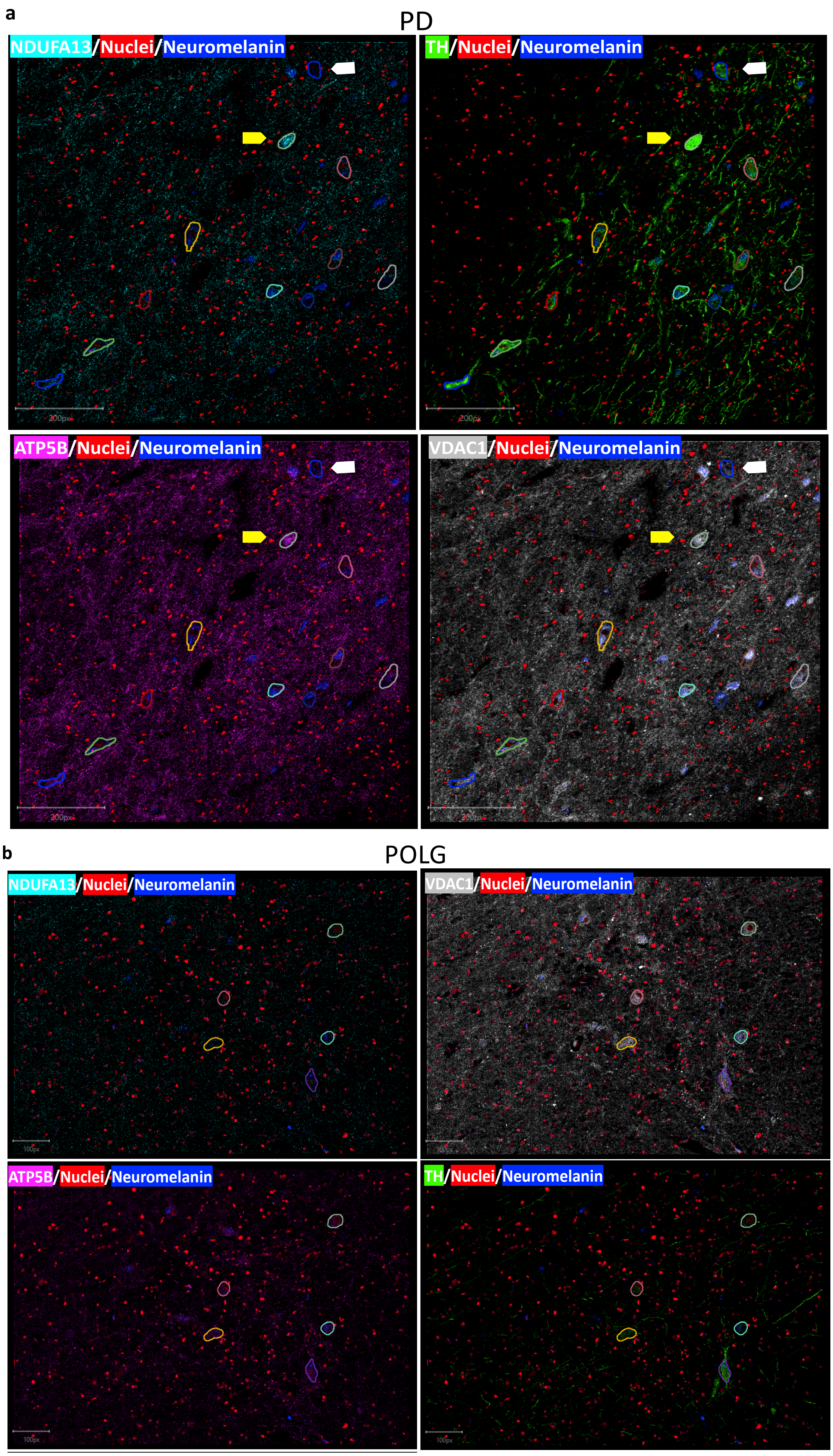
a
PD
NDUFA13/Nuclei/Neuromelanin
TH/Nuclei/Neuromelanin
ATP5B/Nuclei/Neuromelanin
VDAC1/Nuclei/Neuromelanin
b
POLG
NDUFA13/Nuclei/Neuromelanin
VDAC1/Nuclei/Neuromelanin
ATP5B/Nuclei/Neuromelanin
TH/Nuclei/Neuromelanin

c

Control

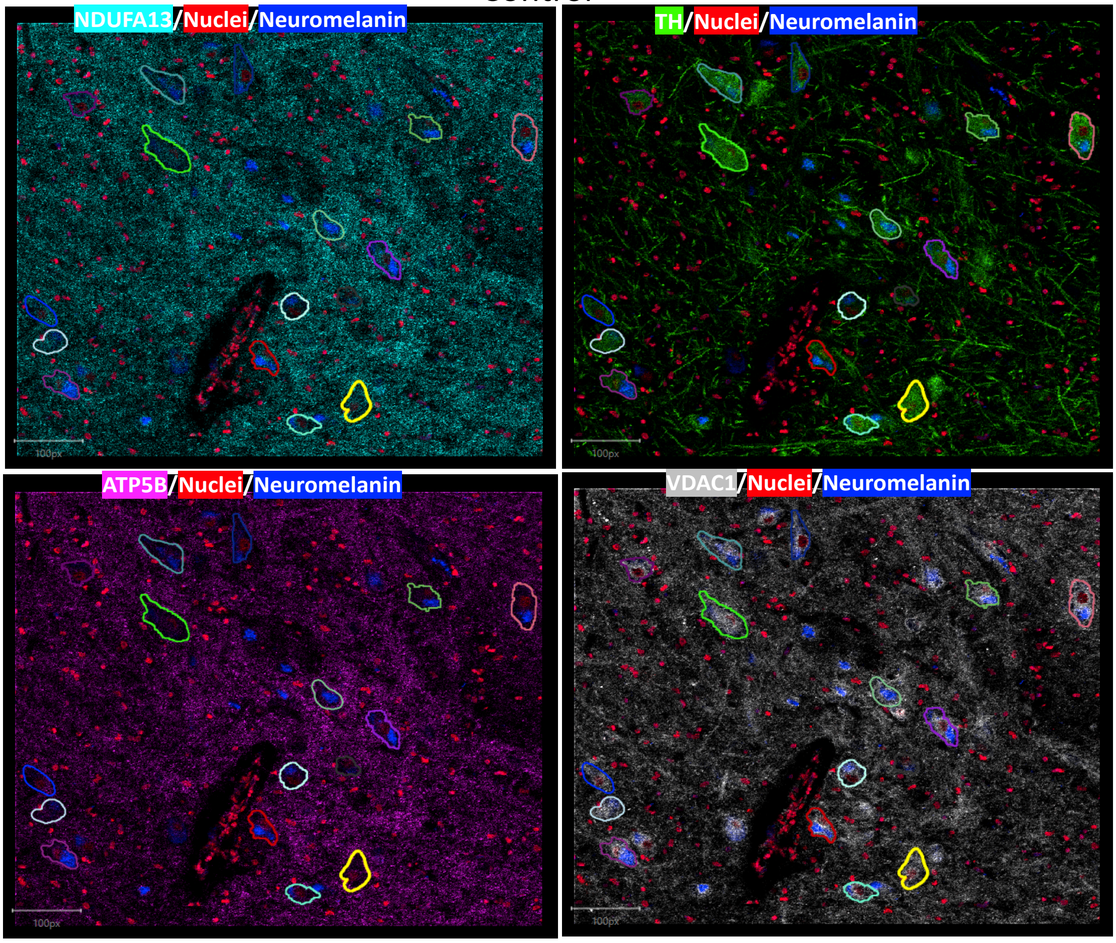

Supplementary Fig. 6. Example ROI images acquired from IMC. Images of three uncropped ROIs from PD03 (a), POLG02(b) and Con05 (c) cases, demonstrating the varying signals of ATP5B and NDUFA13 and the distribution pattern of individual TH+ neurons (outlined). The number and density of selected neurons per demonstrated ROI are as followed: PD03, n=11; 13.4/per mm^2; POLG02, n=5; 6.4/per mm^2; Con05, n=15; 31.5/per mm^2. A neuron showing decreased signal for both ATP5B and NDUFA13 (yellow tag) and another neuron with normal NDUFA13 and ATP5B expression (white tag) was highlighted (a). In the POLG04 ROI (b), the expression signal of NDUFA13 shows a general decrease in the outlined neurons, while most neurons demonstrate an intact ATP5B signal. Scale bar, 100um.

Supplementary table 1. Summary information of the PD and POLG cohorts for this study. PM delay- post mortem delay; PD- Parkinson's disease ; CAA- cerebral amyloid angiopathy; CERAD- Consortium to Establish a Registry for Alzheimer's Disease; LB- Lewy body; Aβ-amyloid beta. POLG02-04 were reported in two previous studies using IF and IHC respectively (POLG3,1,4 in Chen, et al. [7]; POLG2-4 in Reeve, et al. [12].

Case	Sex	Age	Disease duration (years)	PM delay (hours)	Clinical presentation	Lewy Pathology stage	LB Braak Stage	Neuropathology	POLG mutation
PD01	Male	68	9	30	Presented with tremor in the right arm, bradykinesia, rigidity and poor balance.	Entorhinal	I	Brain with α-synuclein pathology. Allocortical tau pathology; Mild iso and allocortical amyloid pathology; Mild meningeal CAA in the occipital lobe.	N/A
PD02	Male	70	15	48	PD without dementia; No cognitive impairment.	Limbic	IV	Brain with subcortical α-synuclein pathology and with tau pathology mainly in the allocortex, without any Aβ pathology except for very mild meningeal CAA in the occipital lobe.	N/A
PD03	Male	80	5	88	PD without dementia, pronounced autonomic features particularly postural hypotension.	Limbic	IV	Limbic Lewy body disease with mild allocortical tau pathology.	N/A
PD04	Male	80	10	34	Tremor, dyskinesia, drooling, postural hypotension, vivid nightmares and visual hallucinations at times	Neocortical	VI	Neocortical Lewy body disease	N/A
PD05	Male	81	12	13	Loss of balance, tremor, some bradykinesia at onset	Neocortical	VI	Brain with α-synuclein pathology. Neocortical Lewy body disease with additional tau pathology mainly restricted to the limbic system.	N/A
PD06	Male	82	11	7	Cognitively intact 3 months before death. Tremor dominant PD	Neocortical	VI	Lewy body disease. Alzheimer type pathology restricted to medial temporal lobe	N/A
PD07	Male	83	6	30	Bilateral tremor with rigidity and bradykinesia, some shuffling, mild postural instability, mild hypomimia.	Brain Stem	III	Brainstem type of Lewy body disease. Mild allocortical tau pathology restricted to the entorhinal cortex.	N/A

PD08	Male	84	9	80	Bradykinesia, unilateral rest tremor.	Limbic	IV	Brain with α-synuclein pathology restricted to the brain stem and cingulate. Limbic transitional lewy bodies disease. Mild Aβ pathology in iso- and allocortex and tau pathology restricted to the entorhinal cortex.	N/A
PD09	Male	90	11	18	Tremor dominant PD	Limbic/brainstem	IV	Brain with α-synuclein pathology in mainly brainstem and limbic regions without any amyloid pathology. Tau pathology mainly in the entorhinal region.	N/A
POLG01	Female	22	5	32	Mitochondrial disease presented as headaches, visual aura- focal and generalised seizures- left hemianopia, bilateral ptosis and uncoordinated movement of left side; Liver failure following valproate; Relentless seizures in terminal phase. Pure mitochondrial encephalopathy.	No LB pathology	0	No abnormal intracytoplasmic or neuritic pathological aggregates were detected within the brainstem samples. The basal-medial occipital cortex showed variably proinent vacuolization, gliosis and loss of neurons. The underlying white matter showed loss of myelin staining and astrogliosis. Moderately severe loss of Purkinje cells and associated patchy Bergmann gliosis. No well-defined infarct, haemorrhage or evidence of a primary neurodegenerative disease was found.	*c.1399G>A (p.Ala467Thr) POLG1 mutation.*
POLG02	Female	23	5	83	Mitochondrial disease presented with neurodegeneration, developed seizures and problems with eye movement and coordination at the age of 20yrs; Progressive ataxia with epilepsy and reduced consciousness in terminal phase.	No LB pathology	0	Severe involvement of the dentate cerebellar nucleus and medullary medial vestibular nucleus, less severe involvement of the brainstem nuclei; Focal degeneration and ischaemic like changes in frontal and parietal regions. Extensive microglia activation in the basal ganglia.	*p.Ala467Thr and p.Trp748Ser POLG1 mutation.*
POLG03	Male	59	37	67	Mitochondrial disease presented initially with ptosis at the age of 22yrs which progressed to almost complete opthalmoplegia; sensorimotor neuropathy; **parkinsonism** (age 50 years); corgnitive impairment with increasing dysphagia, dysarhria and dysphonia.	Brainstem	III	Mitochondrial encephalopathy and Lewy body disease; **α-synuclein pathology in the brainstem and subcortical nuclei**; Scattered infarcts in the frontal cortex, caudate nucleus, cerebellar cortex and posterior temporal white matter; Arteriosclerosis in the frontal cortex, temporal deep white matter, periventricular white matter in the frontal and parietal lobe.	*p.Gly848Ser and p.Ser1104Cys POLG1 mutation*

POLG04	Male	79	23	85	MItochondrial disease with multiple deletions. Chronic progressive external opthalmoplegia, dysphagia, previous MIs, marked ptosis requiring surgery.	Limbic	III	Infarct like lesions- right frontal and parietal lobes and globus pallidus. **Lewy body disease brainstem type** (transitional), tau pathology within temporal lobe (Braak Stage IV), CAA typ2, CERAD frequent. Leukoariosis in periventricular and deep white matter, calcification of vessel walls in globus pallidus and choroid plexus, mamillary body pathology (reminiscent of Wernicke's encephalopathy), patchy loss of neurons in inferior olives and purkinje cells in the cerebellar cortex.	*p.Thr251Ile and p.Pro587Leu and p.Ala467Thr POLG1 mutations*

Supplementary table 2. Summary information of control cases for this study. PM delay- post mortem delay; AD-Alzheimer's disease.

Code	Age	Gender	PM delay (hours)	Lewy pathology category	Case description
Con01	18	Female	81	No Lewy Body pathology	No neurodegenerative or cerebrovascular disease
Con02	19	Male	65	No Lewy Body pathology	No neurodegenerative disease or sclerosis in the right entorhinal cortex and hippocampus
Con03	60	Male	60	No Lewy Body pathology	Presence of very mild age associated amyloid and tau pathology; No parkinsonism
Con04	71	Female	43	No Lewy Body pathology	No neurodegenerative disease
Con05	71	Female	72	No Lewy Body pathology	Presence of very mild age associated amyloid pathology and practically no tau pathology; No parkinsonism
Con06	81	Male	34	No Lewy Body pathology	Presence of age associated amyloid and tau pathology; No parkinsonism
Con07	88	Male	23	No Lewy Body pathology	Presence of tau pathology limited to the entorhinal cortex, hippocampus and locus coeruleus and amyloid pathology extending to the entorhinal cortex; No parkinsonism
Con08	88	Male	69	No Lewy Body pathology	Intermediate AD pathology; No parkinsonism
Con09	90	Male	80	No Lewy Body pathology	Minimal AD neuropathology. No parkinsonism
Con10	93	Female	34	No Lewy Body pathology	Minimal AD neuropathology; No parkinsonism

Supplementary table 3. Output from the Bayesian Estimation for Fig 4. µ1-the Difference of Mean (diseased neurons), µ2- the Difference of Mean (control neurons), HDI- 95% Highest Density Interval; ROPE-Region of Practical Equivalence.

POLG vs. Control								
**Target**	**µ1**	**µ2**	**µ1- µ2**	**HDI low**	**HDI up**	**ROPE low**	**ROPE high**	**%InROPE**
Complex I-NDUFB8	-1.51	-0.02	-1.49	-1.69	-1.30	-0.10	0.10	0.00
Complex I-NDUFA13	-2.32	0.04	-2.36	-2.55	-2.18	-0.10	0.10	0.00
Complex II-SDHA	-0.89	0.05	-0.94	-1.19	-0.70	-0.10	0.10	0.00
Complex III-UqCRC2	-1.30	0.10	-1.39	-1.67	-1.12	-0.10	0.10	0.00
Complex IV-MTCO1	-1.11	0.03	-1.14	-1.35	-0.91	-0.10	0.10	0.00
Complex IV-COXIV	-1.45	0.04	-1.49	-1.72	-1.26	-0.10	0.10	0.00
Complex V-ATP5B	-1.80	0.02	-1.83	-2.10	-1.56	-0.10	0.10	0.00
Complex V-OSCP	-0.16	0.03	-0.19	-0.47	0.09	-0.10	0.10	23.86
Mitomass-VDAC1	-0.31	0.00	-0.30	-0.48	-0.13	-0.10	0.10	1.07
**PD vs. Control**								
Complex I-NDUFB8	-0.25	-0.01	-0.24	-0.41	-0.07	-0.10	0.10	5.42
Complex I-NDUFA13	-0.31	0.04	-0.35	-0.51	-0.20	-0.10	0.10	0.06
Complex II-SDHA	-0.40	0.05	-0.45	-0.62	-0.28	-0.10	0.10	0.00
Complex III-UqCRC2	-0.37	0.09	-0.45	-0.61	-0.30	-0.10	0.10	0.00
Complex IV-MTCO1	-0.26	0.03	-0.29	-0.45	-0.14	-0.10	0.10	0.71
Complex IV-COXIV	-0.53	0.04	-0.57	-0.72	-0.42	-0.10	0.10	0.00
Complex V-ATP5B	-0.85	0.02	-0.87	-1.04	-0.71	-0.10	0.10	0.00
Complex V-OSCP	-0.53	0.03	-0.56	-0.73	-0.40	-0.10	0.10	0.00
Mitomass-VDAC1	-0.22	0.00	-0.21	-0.37	-0.06	-0.10	0.10	7.16

Supplementary table 4. Output from the Bayesian Estimation for Fig 6. Diff_mean_ VDAC1: μ1- μ2; μ1-the Difference of Mean (diseased neurons), μ2- the Difference of Mean (control neurons), HDI- 95% Highest Density Interval; ROPE-Region of Practical Equivalence.

**Complex I**	**caseno**	**Diff_mean**	**ROPE%**	**Effect size**	**Complex II**	**caseno**	**Diff_mean**	**ROPE%**	**Effect size**
	Ctrl04	0.25	11.11	0.26		Ctrl06	0.55	2.92	0.96
	Ctrl05	0.22	19.52	0.23		Ctrl09	0.3	9.63	0.29
	Ctrl06	0.15	24.83	0.2		Ctrl10	0.87	1.89	1.12
	Ctrl08	0.49	7.79	0.71		PD02	0.14	29.7	0.21
	Ctrl10	0.55	3.78	0.73		PD03	-2	10.96	0.11
	PD02	0.14	25.79	0.19		PD04	1.15	0.47	2.2
	PD04	0.56	7.27	0.47		PD07	0.42	9.03	0.69
	PD05	0.39	11.68	0.43		PD08	-0.21	0.1	0.02
	PD06	0.75	6.25	0.38		PD10	-0.25	15.15	0.24
	PD07	0.17	26.63	0.28					
	PD08	0.52	8.57	0.54					
	PD10	0.31	15.7	0.37					
	POLG04	-0.11	38.11	0.21					

**Complex III**	**caseno**	**Diff_mean**	**ROPE%**	**Effect size**	**Complex IV**	**caseno**	**Diff_mean**	**ROPE%**	**Effect size**
	Ctrl05	-0.16	14.3	0.21		Ctrl03	-0.32	11.85	0.47
	Ctrl06	0.61	1.37	1		Ctrl04	-0.15	20.32	0.15
	Ctrl10	0.84	1.39	1.06		Ctrl05	0.41	10.03	0.54
	PD02	-1.08	0.04	2.4		Ctrl06	0.11	11.49	0.09
	PD04	1.01	1.42	1.92		Ctrl08	-0.07	25.76	0.11
	PD05	0.99	1.59	1.11		Ctrl09	2	6.88	0.58
	PD07	0.2	23.03	0.33		Ctrl10	0.38	10.59	0.51
	PD08	0.04	14.16	0.05		PD02	0.12	31.57	0.18

	PD10	0.22	17.39	0.21		PD04	0.47	8.95	0.6
	POLG01	0.16	24.13	0.17		PD05	0.54	8.2	0.48
	POLG03	-0.17	16.95	0.16		PD07	0.39	7.85	0.73
	POLG04	0.34	11.33	0.29		PD08	0	13.33	0.11
						PD10	0.03	30.28	0.07

**Complex V**	**caseno**	**Diff_mean**	**ROPE%**	**Effect size**
	Ctrl02	-0.15	31.29	0.29
	Ctrl04	0.23	16.74	0.25
	Ctrl06	0.49	4.12	0.81
	Ctrl10	0.39	10.6	0.48
	PD02	-0.38	7.5	0.63
	PD03	-0.19	22.75	0.41
	PD04	-0.16	26.72	0.24
	PD05	-0.43	10.93	0.43
	PD07	0.05	16.97	0.07
	PD08	-0.36	3.66	0.88
	PD10	-0.05	27.83	0.06
	POLG04	-0.2	23.34	0.33
