# Supplementary material for: Imaging mass cytometry reveals generalised deficiency in OXPHOS complexes in Parkinson’s disease
Source: NPJ Parkinsons Dis. 2021 May 12;7:39. doi: 10.1038/s41531-021-00182-x (PMC8115071; doi:10.1038/s41531-021-00182-x)
Supplement: Supplementary file 2 — Reporting Summary [file 41531_2021_182_MOESM2_ESM.pdf]

nature research

Corresponding author(s): Amy Reeve

Last updated by author(s): Mar 20, 2021

Reporting Summary

Nature Research wishes to improve the reproducibility of the work that we publish. This form provides structure for consistency and transparency in reporting. For further information on Nature Research policies, see our Editorial Policies and the Editorial Policy Checklist.

Please do not complete any field with "not applicable" or n/a. Refer to the help text for what text to use if an item is not relevant to your study.
For final submission: please carefully check your responses for accuracy; you will not be able to make changes later.

Statistics

For all statistical analyses, confirm that the following items are present in the figure legend, table legend, main text, or Methods section.

n/a	Confirmed	
☐	☒	The exact sample size (*n*) for each experimental group/condition, given as a discrete number and unit of measurement
☐	☒	A statement on whether measurements were taken from distinct samples or whether the same sample was measured repeatedly
☐	☒	The statistical test(s) used AND whether they are one- or two-sided<br>*Only common tests should be described solely by name; describe more complex techniques in the Methods section.*
☐	☒	A description of all covariates tested
☐	☒	A description of any assumptions or corrections, such as tests of normality and adjustment for multiple comparisons
☐	☒	A full description of the statistical parameters including central tendency (e.g. means) or other basic estimates (e.g. regression coefficient) AND variation (e.g. standard deviation) or associated estimates of uncertainty (e.g. confidence intervals)
☐	☒	For null hypothesis testing, the test statistic (e.g. F, t, r) with confidence intervals, effect sizes, degrees of freedom and P value noted<br>*Give P values as exact values whenever suitable.*
☒	☐	For Bayesian analysis, information on the choice of priors and Markov chain Monte Carlo settings
☒	☐	For hierarchical and complex designs, identification of the appropriate level for tests and full reporting of outcomes
☐	☒	Estimates of effect sizes (e.g. Cohen's *d*, Pearson's *r*), indicating how they were calculated

Our web collection on statistics for biologists contains articles on many of the points above.

Software and code

Policy information about availability of computer code

Data collection	Images generated from IMC were exported as tiffs (MCD viewer, Fluidigm) and converted to pseudocoloured images for visualisation using Fiji and QuPath 0.2.0
Data analysis	Statistical analysis and graph generation was performed using R 3.6.1, the ggplot2 package and the BEST package

For manuscripts utilizing custom algorithms or software that are central to the research but not yet described in published literature, software must be made available to editors and reviewers. We strongly encourage code deposition in a community repository (e.g. GitHub). See the Nature Research guidelines for submitting code & software for further information.

Data

Policy information about availability of data

All manuscripts must include a data availability statement. This statement should provide the following information, where applicable:
- Accession codes, unique identifiers, or web links for publicly available datasets
- A list of figures that have associated raw data
- A description of any restrictions on data availability

Raw datasets generated and analysed during the current study(for all figures and supplementary materials) are available in the ‘Figshare' repository (doi: 10.25405/data.ncl.14079497). Data are available under the terms of the Creative Commons Attribution 4.0 International license (CC-BY 4.0). The raw datasets are available for all figures and supplementary materials.

Field-specific reporting

Please select the one below that is the best fit for your research. If you are not sure, read the appropriate sections before making your selection.

- [x] Life sciences
- [ ] Behavioural & social sciences
- [ ] Ecological, evolutionary & environmental sciences

For a reference copy of the document with all sections, see nature.com/documents/nr-reporting-summary-flat.pdf

Life sciences study design

All studies must disclose on these points even when the disclosure is negative.

Sample size

The number of cases included in each group was based on the number included in previous studies using post-mortem samples. All neurons within the substantia nigra were analysed from one section from each case (lsted in Table 3 in the manuscript). Individual neuronal number varies from case to case due to this sampling method and the presence of neurodegeneration in some cases.

Data exclusions

No data were excluded from analysis

Replication

This study aimed to validate a novel technique for use in FFPE brain tissue and provide profiling of mitochondrial protein expression. Multiple neurons from each individual were taken and this was replicated across a number of individuals. Several of the protein targets included here have been included in further, ongoing studies allowing further replication of these experiments.

Randomization

Samples were grouped based on presence of neurodegenerative disease and the type of disease. Three groups were studied, healthy controls (with no neurological disease), cases with Parkinson's disease and cases with mitochondrial disease caused by POLG mutations. This grouping is appropriate given that we wanted to study the differences between these three groups to understand more about the changes specific to PD.

Blinding

Investigators were blinded to disease group during data collection and allocations only revealed when required for analysis. Case numbers were used for blinding.

Reporting for specific materials, systems and methods

We require information from authors about some types of materials, experimental systems and methods used in many studies. Here, indicate whether each material, system or method listed is relevant to your study. If you are not sure if a list item applies to your research, read the appropriate section before selecting a response.

Materials & experimental systems

n/a	Involved in the study	
[ ]	[x]	Antibodies
[x]	[ ]	Eukaryotic cell lines
[x]	[ ]	Palaeontology and archaeology
[x]	[ ]	Animals and other organisms
[x]	[ ]	Human research participants
[x]	[ ]	Clinical data
[x]	[ ]	Dual use research of concern

Methods

n/a	Involved in the study	
[x]	[ ]	ChIP-seq
[x]	[ ]	Flow cytometry
[x]	[ ]	MRI-based neuroimaging

Antibodies

Antibodies used

Anti-NDUFB8 (Clone: 20E9DH10C12; RRID: AB_10859122) Complex I (nDNA encoded subunit) ab218218 Abcam
Anti-NDUFA13 (Clone: 6E1BH7;RRID: AB_10863178) Complex I (nDNA encoded subunit) ab218217 Abcam
Anti-SDHA (Clone: 2E3GC12FB2AE2; RRID: AB_301433)) Complex II (nDNA encoded subunit) ab218213 Abcam
Anti-UqCRC2 (Clone: 13G12AF12BB11; RRID: AB_2213640) Complex III (nDNA encoded subunit) ab218215 Abcam
Anti-MTCO1 (Clone: 1D6E1A8; RRID: AB_2084810) Complex IV (mtDNA encoded subunit) ab218212 Abcam
Anti-COX4+4L2 (Clone: 10G8D12C12; RRID: AB_10862891) Complex IV (nDNA encoded subunit) ab218231 Abcam
Anti-ATB5B (Clone: 3D5; RRID: AB_301438) Complex V (nDNA encoded/ core subunit) ab14730 Abcam
Anti-ATP5O/OSCP (Clone: 4C11C10D12; RRID: AB_10887942) Complex V (nDNA encoded/ rotary stalk) ab218232 Abcam
Anti-VDAC1 (Clone: 20B12AF2; RRID: AB_443084) Mito mass marker ab218214 Abcam
Anti-TH (Clone: TH-16; RRID: AB_477569) Dopaminergic marker SAB4200697 Sigma
Anti-Histone H3-171Yb (Clone: D1H2; RRID: AB_2811058) Nuclear marker 3176023D, Fluidigm

Validation

Describe the validation of each primary antibody for the species and application, noting any validation statements on the manufacturer's website, relevant citations, antibody profiles in online databases, or data provided in the manuscript.

This checklist template is licensed under a Creative Commons Attribution 4.0 International License, which permits use, sharing, adaptation, distribution and reproduction in any medium or format, as long as you give appropriate credit to the original author(s) and the source, provide a link to the Creative Commons license, and indicate if changes were made. The images or other third party material in this article are included in the article's Creative Commons license, unless indicated otherwise in a credit line to the material. If material is not included in the article's Creative Commons license and your intended use is not permitted by statutory regulation or exceeds the permitted use, you will need to obtain permission directly from the copyright holder. To view a copy of this license, visit http://creativecommons.org/licenses/by/4.0/
